# Factors associated with desired fertility among HIV-positive women and men attending two urban clinics in Lilongwe, Malawi

**DOI:** 10.1371/journal.pone.0198798

**Published:** 2018-06-13

**Authors:** Jamie W. Krashin, Lisa B. Haddad, Hannock Tweya, Jane Chiwoko, Wingston Ng’ambi, Bernadette Samala, Thomas Chaweza, Jennifer H. Tang, Mina C. Hosseinipour, Sam Phiri

**Affiliations:** 1 Department of Gynecology and Obstetrics, Emory University School of Medicine, Atlanta, Georgia, United States of America; 2 Department of Obstetrics & Gynecology, University of North Carolina School of Medicine, Chapel Hill, North Carolina, United States of America; 3 The Lighthouse Trust, Kamuzu Central Hospital, Lilongwe, Malawi; 4 UNC Project-Malawi, Kamuzu Central Hospital, Lilongwe, Malawi; 5 Department of Medicine, University of North Carolina School of Medicine, Chapel Hill, North Carolina, United States of America; The Ohio State University, UNITED STATES

## Abstract

As access to antiretroviral therapy increases, more HIV-infected patients in sub-Saharan Africa may desire fertility. We conducted a cross-sectional study of reproductive health knowledge, attitudes and practices to identify factors associated with desired fertility among women and men receiving care at two large public HIV clinics in Lilongwe, Malawi. Research assistants administered questionnaires to participants. We performed descriptive, bivariable and multivariable analysis of factors related to desired fertility and of factors related to contraceptive non-use among participants who did not desire fertility. One-third of participants desired future children. Having a partner who desired fertility and having lower parity were associated with desiring children among both genders. For women, believing that pregnancy was unhealthy was associated with decreased fertility desire. Fifty-five percent of women and 69% of men who did not want children in the future reported using contraception at last intercourse. Increasing age, lower parity, and making the decision to use contraception herself were associated with contraceptive non-use among women who did not desire fertility. Having discussed family planning with his partner was associated with contraceptive use among men who did not desire fertility. Knowledge of these factors can guide reproductive health counseling and service provision.

## Introduction

As more people access antiretroviral therapy (ART) and live healthier and longer lives with human immunodeficiency virus (HIV) in sub-Saharan Africa, their desire for future pregnancy may be increasing [1–6]. HIV prevalence and ART use in Malawi is similar to much of sub-Saharan Africa: 10.6% of reproductive-aged adults are HIV-positive, and two-thirds of those eligible receive ART [7]. Among people living with HIV in sub-Saharan Africa, 35–75% desire children in the future [2,3,8–16]. In addition, people living with HIV, especially reproductive-age women on ART, tend to access health care more regularly than their peers [7]. HIV-positive women and men are a large and accessible group in need of reproductive life planning discussions [17]. These discussions would allow for targeted provision of contraception and medical guidance for achieving the healthiest possible pregnancy.

The desire for future childbearing among people living with HIV in sub-Saharan Africa has been associated with several sociodemographic, reproductive history, cultural, and HIV-related factors. Younger age, fewer living children, and having a partner who desires fertility have been consistently associated with increased desire to have future children among HIV-positive women and men in different populations [4,10–13,15, 18–20]. To date, these factors associated with desired fertility have not been studied among women and men receiving care at large public HIV clinics in Malawi. This report identifies factors associated with desired fertility among HIV-positive women and men receiving care at large public HIV clinics in Lilongwe, Malawi’s capital. We also sought to describe contraceptive use and factors associated with not using contraception among participants who did not desire future fertility.

## Materials and methods

### Participants and procedures

We conducted a cross-sectional study of reproductive health knowledge, attitudes and practices among men and women receiving HIV-related care in Lilongwe, Malawi. The National Health Services Research Committee in Malawi and the institutional review boards at Emory University and University of North Carolina-Chapel Hill formally approved the study. All participants provided written informed consent prior to participation.

Women and men were recruited at two Lighthouse Trust clinics in Lilongwe, Malawi from September 26, 2013 to December 20, 2013. The Lighthouse Trust is a Centre of Excellence for Integrated HIV Prevention, Treatment and Care; and provided care for over 23,000 patients on ART and over 2,000 patients who were not yet clinically eligible for ART by December 2013. The Lighthouse Clinic is at Kamuzu Central Hospital, a tertiary referral hospital for the central region of Malawi, and the Martin Preuss Centre is at Bwaila Hospital falling under the Lilongwe District Hospital. Both are public clinics offering HIV treatment in the capital, and both began offering family planning services in 2010.

Study staff recruited patients by consecutive, non-random sampling from the clinic waiting areas and screened interested patients in private. We planned to enroll a convenience sample of 300 women and 300 men over a three-month period, based on budget and historical clinic attendance. Eligibility criteria included ages 18–45 years, fluency in Chichewa (the most commonly spoken local language), having a sexual partner within the past six months, having documented HIV-positive status, and receiving care at either Lighthouse clinic or Martin Preuss Centre. Research assistants administered the questionnaire in Chichewa. Male and female participants may have had partners who completed the study, but they were not interviewed as couples.

#### Survey and measures

The questionnaire included 160 questions for women and 130 for men (S1 and S2 Text). Research questions were developed through a combination of focus group discussions among female and male patients at the Lighthouse clinics and questions used in the Malawi 2010 Demographic and Health Survey [21]. All data from the questionnaires were double entered into a Microsoft Access 2007 (Microsoft, Redmond, WA, USA) database and validated using predetermined queries.

This report addresses one aspect of the study’s primary objective: desired fertility. This outcome was measured by a dichotomous “yes/no” answer the question: “Do you want or plan to have more children (at any time in the future)?” Potential variables related to fertility intention fell into six categories: sociodemographic, HIV-related, sexual characteristics and risk behaviors, reproductive history, communication and social pressure, and perceived risk of HIV in pregnancy. We also asked participants if they were currently using contraception and what method(s) of contraception they or their partners used at last intercourse.

### Statistical analysis

We conducted descriptive, bivariable and multivariable analyses of variables potentially associated with the desire to have children in the future and contraceptive use among those who did not desire fertility. The descriptive analysis included participant characteristics and information on reported contraceptive use. We used Pearson’s χ^2^ and Fisher’s exact where appropriate to test bivariable associations. To describe unmet need for contraception in this group, we determined the frequency of current contraceptive use and reported types of contraception used by participants who did not desire fertility.

We planned separate regression models for each gender. The maximum number of variables included in each gender’s final model was limited to *m*/10, where *m* is the smaller of the number of participants either desiring or not desiring future children. We first identified potential variable associated with desired fertility and contraceptive use in the literature and consensus clinical experience. From this list, we then eliminated any variables that likely duplicated the same construct, had a prevalence of <10% in one of the outcomes, or were less consistently associated with our outcome in the literature to reach the acceptable number of variables for each model. We ran Poisson regressions with robust standard errors to estimate unadjusted and adjusted prevalence ratios with 95% confidence intervals [22]. We used Stata (Release 13, StataCorp, College Station, TX, USA) for statistical analysis.

## Results

Between September and December 2013, we screened 349 women and 274 men, and enrolled 308 (88%) and 254 (93%), respectively, for a total of 562 participants. Fewer men attended clinic during these 3 months than anticipated when planning our convenience sample. Of the 562 participants enrolled, 308 women and 250 men had data on our primary outcome and are included in this report (S1 Fig).

Thirty-four percent (105/308) of women and 28% (70/254) of men desired fertility (Table 1). Of participants who did not want future children, 101/204 (50%) women and 76/180 (42%) men desired fertility if they were counterfactually HIV negative (S1 Table).

**Table 1:** Characteristics of HIV-positive women and men who received care at large, public HIV clinics in Lilongwe, Malawi.

Characteristic		Total (n = 558) n (%)	Women (n = 308) n (%)	Men (n = 250) n (%)
Desires fertility	Yes	175 (31)	105 (34)	70 (28)
Sociodemographic				
Age	18–25 years	52 (9)	48 (15)	4 (2)
	26–35 years	252 (45)	159 (52)	93 (37)
	36–40 years	110 (20)	52 (17)	58 (23)
	Over 40 years	144 (26)	49 (16)	95 (38)
Urban residence (n = 307 women)		489 (88)	276 (90)	213 (85)
Education: completed secondary school		107 (19)	51 (17)	56 (22)
Religion (n = 307 women, n = 249 men)	Catholic	113 (20)	62 (20)	51 (20)
	Protestant	395 (71)	220 (72)	175 (70)
	Muslim	43 (8)	24 (8)	19 (8)
	None	5 (<1)	1 (<1)	4 (2)
HIV-related				
Years since HIV diagnosis (n = 305 women)	< 1 year	75 (14)	30 (10)	45 (18)
	1 to <5 years	229 (41)	129 (42)	100 (40)
	5 to <10 years	214 (38)	130 (43)	84 (34)
	≥10 years	37 (7)	16 (5)	21 (8)
Currently taking ART	Yes	492 (88)	273 (89)	219 (88)
Health status since beginning ART^a^	Improved	457 (93)	252 (92)	205 (94)
	Worsened	30 (6)	18 (7)	12 (5)
	No change	5 (1)	3 (1)	2 (1)
Sexual characteristics and risk behaviors				
Married or in monogamous relationship		546 (98)	300 (97)	246 (98)
Most recent partner’s HIV status (n = 307 women, n = 249 men)	Positive	378 (68)	195 (63)	183 (73)
	Negative	102 (18)	58 (19)	44 (18)
	Unknown	76 (14)	54 (18)	22 (9)
Length of time with current partner (n = 300 women, n = 247 men)	< 4 years	174 (32)	99 (33)	75 (30)
	> 4 years	373 (68)	201 (67)	172 (70)
Who decides whether to use contraception (n = 285 women, n = 223 men)	Woman does	292 (58)	203 (71)	89 (40)
	Man does or both do	192 (42)	80 (28)	134 (60)
Reproductive history				
Number of children	0	28 (5)	23 (7)	5 (2)
	1	105 (19)	65 (21)	40 (16)
	2–3	259 (46)	146 (48)	113 (45)
	4 or more	166 (30)	74 (24)	92 (37)
Has child born with HIV (n = 307 women)		452 (81)	247 (80)	205 (82)
Most recent partner desires fertility (n = 305 women, n = 249 men)		196 (35)	128 (42)	68 (27)
Communication and social pressure				
Discussed fertility desires or family planning as a couple		487 (87)	258 (84)	229 (92)
Disclosed HIV status to most recent partner (n = 249 men)		525 (94)	288 (94)	237 (95)
Believes there is pressure for women to have children, regardless of HIV status		490 (88)	278 (90)	212 (85)
Feels pressure to have children from family or community		161 (29)	94 (31)	67 (27)
Discouraged from childbearing by healthcare worker when HIV status was disclosed^b^			44 (14)	n/a
Believes physician would support her decision to have (more) children^b^			177 (57)	n/a
HIV and pregnancy				
Believes HIV-positive women can give birth to HIV-negative babies (n = 307 women)		537 (96)	299 (97)	238 (95)
Perceived effect of ART on risk of MTCT^b^ (n = 305 women)	Increased risk		94 (31)	n/a
	Decreased risk		185 (61)	
	No change		26 (8)	
Believes pregnancy is unhealthy for her^b^			200 (65)	n/a

ART = antiretroviral therapy, MTCT = mother-to-child-transmission

^a^ Percentages of participants on ART (n = 273 women and n = 219 men)

^b^ Questions asked of female participants only

Participant ages spanned the reproductive spectrum, and the mean ages of participants were 32 (SD 6.5) years for women and 37 (SD 5.4) years for men. The median time since being diagnosed with HIV was 4.0 years (IQR 2–7) for women and 3.5 (IQR 1–7) years for men. Most participants were taking ART, had at least one child, and did not feel pressured by family or community to have future children. Approximately one-fifth of participants had a child born with HIV. Forty-two percent (128/308) of women and 70% (68/250) of men had a partner who desired fertility. A small proportion (14%, 44/302) of women were discouraged from childbearing by healthcare workers who knew their status, and more than half believed that a physician would support her decision to have future children. Although nearly all participants knew that HIV-positive women could give birth to HIV-negative infants, almost a third of women thought that using ART in pregnancy increased the risk of transmitting HIV to infants. Approximately two-thirds of women believed that pregnancy would be unhealthy for them.

In unadjusted analyses of women, lower parity, partner desiring fertility, and pressure from family or community were associated with increased fertility desire (Table 2). Older age, having a child born with HIV, and believing that pregnancy was unhealthy were associated with decreased fertility desire. In the multivariable analysis for women, lower parity and partner desiring fertility remained significantly associated with the desire for future pregnancy. Believing pregnancy to be unhealthy for her also remained associated with decreased fertility desire. The strongest association was with partner desiring fertility (aPR 4.18, 95% CI 2.69–6.47).

**Table 2:** Unadjusted and adjusted prevalence ratios of HIV-positive women and men who desire children in the future and who received care at large, public HIV clinics in Lilongwe, Malawi.

		Women		Men	
Variable		Unadjusted PR (95% CI)	Adjusted PR (95% CI) n = 301	Unadjusted PR (95% CI)	Adjusted PR (95% CI) n = 249
Age^a^		0.94 (0.92–0.96)	0.98 (0.96–1.01)	0.94 (0.91–0.98)	0.98 (0.95–1.01)
Time since HIV diagnosis^a^		0.95 (0.91–1.00)	1.01 (0.97–1.06)	1.03 (0.99–1.08)	1.03 (1.00–1.07)
Currently using ART		0.71 (0.48–1.06)	1.11 (0.79–1.55)	0.96 (0.53–1.73)	1.29 (0.85–1.94)
Number of children	0–1	4.20 (2.38–7.43)	2.23 (1.30–3.83)	4.38 (2.59–7.41)	2.47 (1.43–4.25)
	2–3	1.80 (0.98–3.30)	1.52 (0.92–2.51)	1.51 (0.84–2.73)	1.83 (1.10–3.04)
	≥4	Ref	Ref	Ref	Ref
Partner desires fertility		5.53 (3.63–8.43)	4.18 (2.70–6.47)	5.32 (3.51–8.07)	4.60 (2.94–7.19)
Pressured by family or community to have future children		2.15 (1.60–2.89)	1.01 (0.77–1.32)	1.82 (1.23–2.69)	1.14 (0.83–1.59)
History of child born with HIV		0.58 (0.35–0.97)	0.70 (0.46–1.06)	1.24 (0.78–1.99)	1.20 (0.82–1.76)
Believes pregnancy is unhealthy for her		0.42 (0.30–0.55)	0.61 (0.46–0.80)	n/a	n/a
Believes physician would support her decision to have children		1.36 (0.98–1.89)	1.11 (0.85–1.45)	n/a	n/a

PR = prevalence ratio; aPR = adjusted prevalence ratio, CI = confidence interval; ART = antiretroviral therapy

^a^ for each increasing year

In unadjusted analyses of men, younger age, having fewer children, partner desiring fertility, and pressure from family or community were associated with desired fertility (Table 2). In the multivariable analysis, partner desiring fertility and having fewer children were significantly associated with the desire for future pregnancy. Partner desire was again the strongest association (aPR 4.60, 95% CI. 2.94–7.18).

Among participants who did not desire fertility, 55% of women (111/202) and 69% of men (125/180) used contraception at last intercourse (Table 3). Only 18% (37/202) of women and 17% (24/180) of men used dual protection. Thirty-eight percent (42/202) of women used a highly effective contraceptive method (>99% effective, including implant, intrauterine contraception, tubal ligation or vasectomy), although 96% (198/202) used a method that was at least moderately effective (>90% effective, including highly effective methods as well as depot medroxyprogesterone acetate or oral contraceptives). Among men, 19% (35/180) used a highly effective method and 42% (75/180) used at least a moderately effective method.

**Table 3:** Contraceptive use relied on at last intercourse among HIV-positive women and men who do not want to have children in the future and who are receiving care at large, public HIV clinics in Lilongwe, Malawi.

		Women (n = 202)^a^ n (%)	Men (n = 180) n (%)
Used contraception at last intercourse		111 (55)	125 (69)
Dual method use at last intercourse ^b^		37 (33)	30 (24)
Most effective method used at last intercourse	Implant	26 (23)	21 (17)
	Vasectomy	0 (0)	13 (10)
	Intrauterine contraception	1 (1)	1 (1)
	Tubal ligation	15 (14)	0 (0)
	Depot medroxyprogesterone acetate	57 (51)	31 (25)
	Oral contraceptives	8 (7)	9 (7)
	Condoms	3 (3)	49 (39)
	Withdrawal	1 (1)	1 (1)

^a^ missing data on contraceptive use for 1 woman who did not desire fertility

^b^ condoms with another contraceptive method

In unadjusted analyses of women who did not desire fertility, lower parity and the woman deciding to use contraception on her own were associated with increased contraceptive non-use (Table 4). In the multivariate analysis, these factors as well as increasing age were associated with contraceptive non-use. The strongest association, which had a two-fold increase prevalence of contraceptive non-use, was having zero to one children compared with at least four children.

**Table 4:** Unadjusted and adjusted prevalence ratios of contraceptive non-use by HIV-positive women and men who do not desire fertility and who receive care at large, public HIV clinics in Lilongwe, Malawi.

		Women		Men	
Variable		Unadjusted PR (95% CI)	Adjusted PR (95% CI) n = 181	Unadjusted PR (95% CI)	Adjusted PR (95% CI) n = 180
Age^a^		1.03 (1.00–1.06)	1.05 (1.02–1.09)	0.97 (0.93–1.01)	0.97 (0.92–1.01)
Number of children	0–1	1.75 (1.18–2.60)	2.16 (1.32–3.53)	1.23 (0.55–2.77)	0.98 (0.45–2.15)
	2–3	1.11 (0.76–1.64)	1.16 (0.80–1.70)	1.23 (0.77–1.99)	1.08 (0.64–1.83
	≥4	Ref	Ref	Ref	Ref
Currently taking ART		1.25 (0.68–2.30)	1.45 (0.66–3.22)	1.39 (0.62–3.12)	1.41 (0.62–3.20)
Disclosed HIV status to most recent partner		0.67 (0.44–1.04)	0.88 (0.50–1.56)	0.44 (0.24–0.81)	0.58 (0.28–1.21)
Woman makes decisions regarding contraceptive use		1.40 (1.02–1.93)	1.47 (1.07–2.03)		
Length of time with current partner		0.89 (0.63–1.26)	0.84 (0.57–1.24)		
Believes pregnancy is unhealthy for her		0.74 (0.54–1.01)	0.76 (0.54–1.07)		
Completed secondary school or more		1.35 (0.96–1.90)	1.39 (0.97–2.01)		
Discussed fertility desires or family planning as a couple				0.43 (0.26–0.72)	0.49 (0.27–0.89)

PR = prevalence ratio; aPR = adjusted prevalence ratio, CI = confidence interval; ART = antiretroviral therapy

^a^ for each increasing year

In unadjusted analyses of men who did not desire fertility, having disclosed his HIV status to his partner and having discussed fertility desires or family planning as a couple were associated with decreased contraceptive non-use (Table 4). In adjusted analysis, men who did not desire fertility but did discuss fertility desires or family planning with their partners had half the prevalence of contraceptive non-use as men who did not discuss these matters with their partners (aPR 0.49, 95% CI: 0.27–0.89).

## Discussion

Our study highlights several critical reproductive health issues that need to be addressed for people living with HIV. A third of participants in our study desired fertility and this was similar for both women and men. Lower parity and having a partner who desired fertility were associated with this desire for both genders. Women who thought that pregnancy was unhealthy were less likely to desire fertility. For those not desiring children, the unmet need for contraception among our study participants was high. These findings raise three important issues. First, addressing desired fertility among HIV-positive patients is important, especially because knowledge of safer conception and prevention of maternal-to-child transmission has been shown to be low in other low and middle income countries [23–25]. Second, clinical factors most easily accessible to providers, such as time since HIV diagnosis and ART use, may be less relevant to patients desiring fertility than reproductive history and personal relationships. Third, decreasing the unmet need for contraception is crucial.

Addressing reproductive life planning for people living with HIV is essential. A third of participants wanted to have children in the future, comparable to HIV-positive women and men in South Africa, Tanzania, Ethiopia, Uganda and among patient in private clinics in Malawi [2,4,8–15]. Asking people living with HIV about their intentions for pregnancy is necessary to improve maternal and child health outcomes, including optimizing other health conditions before conception.

In addition to improving health outcomes, reproductive life planning discussions for people living with HIV, dispelling misconceptions, would provide education on HIV prior to conception. Half the women not desiring fertility would want future children if they were not HIV-positive, and the belief that pregnancy was unhealthy was negatively associated with desiring fertility. More women might want to become pregnant if they were aware that pregnancy does not exacerbate maternal HIV disease if managed with ART and were counseled on how to best achieve a healthy pregnancy and reduce the risk of HIV transmission to their children. Most women in our study knew that HIV-positive women could have HIV-negative babies, but almost a third thought that ART increased the risk of maternal-to-child-transmission (MTCT) of HIV. A woman’s belief in whether pregnancy is unhealthy for her may be similar to HIV treatment optimism and concern about premature death, factors that have been related to the desire for future pregnancies among HIV positive women in the region [26,27]. Responses highlight the prevalence of community misconceptions regarding MTCT, HIV progression, and their possible influence on family planning goals among people living with HIV. Previous studies have outlined additional barriers to implementation of safer conception, including health care provider (HCP) training and HCPs sharing positive experiences with elimination of MTCT and safer conception methods during universal test-and-treat [28,29]. The ability to act on improved knowledge is also crucial: access to ART/PMTCT services in Uganda increased the desire for future childbearing among women [25]. Improved and ongoing counseling alongside increased access to ART may allow couples to better understand their options and act on their desires for family building.

Time since HIV was diagnosed and ART use—information accessible to clinical providers and previously reported as associated with the desire to have children in other studies—were not statistically significant in our adjusted model [2,4,30]. Our findings on ART use may have been affected by the high prevalence of ART use in our population. Studies outside of Malawi have shown mixed results for whether time since HIV diagnosis is related to desire for future children [26,31]. Factors that influence desire for children among uninfected individuals may similarly be influential among HIV-infected men and women.

Similar to other studies in Malawi and other sub-Saharan countries, we found that having fewer children and partner’s desired fertility were associated with HIV-positive men and women desiring fertility [2,4,10–13,19,20,32]. Interestingly, age—which is inconsistently reported as associated with fertility intentions—was not associated with the desire for future children in our study. It is likely that some correlation exists between age and parity, and parity drives the association with desire for children more so than age. In addition, in other studies that analyzed age as a continuous variable, the effect size was small and very similar to ours [4,15,18].

The unmet need for contraception among participants who did not desire fertility in the future was high. Among those using contraception, few used the most effective, longest-acting methods. In particular, only two participants reported using the copper intrauterine device, which is both extremely effective and safe for use for most HIV-infected women. Given that many people living with HIV may want to have children in the future after counseling, increased access to highly effective reversible methods is especially important. Our study clinics offered the range of reversible methods listed in Table 3, and permanent methods could be obtained at the public hospitals where clinics are located. Our findings may therefore not be generalizable to clinics without integrated family planning. Although this study was unable to assess barriers to contraceptive use, evaluation of national, local, and clinic-level barriers to contraceptive access may help HIV clinics and providers remove specific barriers to providing HIV-positive individuals effective and desired contraception.

Understanding factors related to contraceptive non-use among women and men who do not desire fertility may help with addressing the unmet need for contraception. Among women who did not desire fertility, increasing age and the woman making contraceptive decisions by herself were counter-intuitively associated with contraceptive non-use. This may reflect decreased perceived risk of fertility along with increased reproductive autonomy with age. Among men who did not desire fertility, discussing family planning with his partner was associated with contraceptive use. The positive association between discussing contraception with male partners and contraceptive use has been demonstrated previously [33]. Future studies that directly ask this population about reasons for non-use would provide important information to guide contraceptive counseling.

Our results are subject to limitations. Our questionnaire measured both fertility desire and planning together. We chose to focus on desired fertility as our outcome, but participants may have considered the planned intention in their answer. Our convenience sample might not have fully represented the population of patients at the clinics. We enrolled participants prior to changing guidelines regarding injectable contraception among women at high risk for HIV acquisition and studies that raised concern for interactions between certain ART and implant effectiveness [34–36]. Our study population was a mostly parous group of men and women in long-term partnerships who were receiving care at large public HIV clinics with affiliations to academic centers in Malawi and the United States. Our findings may therefore not be generalizable outside of this group. The homogeneity of our participants in regards to ART use, being in committed relationships, and disclosing their status to partners also limited our ability to study these factors previously found to be related to desired fertility [2,10,11,18,19,32]. However, the two clinics provide HIV management for a large portion of HIV-positive people in Malawi’s capital.

## Conclusions

Our findings provide guidance for medical providers and community workers caring for HIV-positive people in Malawi. People living with HIV deserve a full discussion of reproductive life planning. Although the majority of HIV positive patients need access to contraception to meet their reproductive goals, many desire and achieve future childbearing. Knowledge of factors associated with the desire for pregnancy may help frame discussions with this population and address gaps in knowledge about safer conception and prevention of maternal-to-child transmission of HIV. Future studies that evaluate the best way to deliver reproductive life planning to this population are necessary.

## Supporting information

S1 Text Study questionnaire in Chichewa. (DOCX)

S2 Text Study questionnaire in English. (DOCX)

S1 Fig Flow diagram of participants screened for desire for future children among HIV-positive women and men in Malawi. (PPTX)

S1 Table Bivariable characteristics associated with desire for fertility among women and men who receive care at large, public HIV clinics in Lilongwe, Malawi. (DOC)

S2 Table Bivariable characteristics associated with contraceptive use at last intercourse among women and men who receive care at large, public HIV clinics in Lilongwe, Malawi and do not desire fertility. (DOC)

## References

[1] Hoffman IF, Martinson FE, Powers KA, Chilongozi DA, Msiska ED, Kachipapa EI, et al The year-long effect of HIV-positive test results on pregnancy intentions, contraceptive use, and pregnancy incidence among Malawian women. Journal Acuir Immune Defic Syndr. 2008;47(4): 477–83. 10.1097/QAI.0b013e318165dc52 18209677

[2] Kawale P, Mindry D, Stramotas S, Chilikoh P, Phoya A, Henry K, et al Factors associated with desire for children among HIV-infected women and men: a quantitative and qualitative analysis from Malawi and implications for the delivery of safer conception counseling. AIDS Care. 2014;26(6): 769–76. doi: 10.1080/09540121.2013.855294 24191735 10.1080/09540121.2013.855294 PMC3943633

[3] Cooper D, Moodley J, Zweigenthal V, Bekker LG, Shah I, Myer L. Fertility intentions and reproductive health care needs of people living with HIV in Cape Town, South Africa: implications for integrating reproductive health and HIV care services. AIDS Behav. 2009;1(1 Suppl): 38–46. 10.1007/s10461-009-9550-1 19343492

[4] Myer L, Morroni C, Rebe K. Prevalence and determinants of fertility intentions of HIV-infected women and men receiving antiretroviral therapy in South Africa. AIDS Patient Care STDs. 2007;21(4): 278–85. doi: 10.1089/apc.2006.0108 17461723 10.1089/apc.2006.0108

[5] Haddad LB, Feldacker C, Jamieson DJ, Tweya H, Cwiak C, Chaweza T, et al Pregnancy prevention and condom use practices among HIV-infected women on antiretroviral therapy seeking family planning in Lilongwe, Malawi. PloS ONE. 2015;10(3): e0121039 doi: 10.1371/journal.pone.0121039 25811849 10.1371/journal.pone.0121039 PMC4374940

[6] Kaida A, Andia I, Maier M, Strathdee SA, Bangsberg DR, Spiegel J, et al The potential impact of antiretroviral therapy on fertility in sub-Saharan Africa. Curr HIV/AIDS Rep. 2006;3(4): 187–94. 17032579 10.1007/s11904-006-0015-0

[7] Government of Malawi. Malawi AIDS Response Progress Report. 2015. http://www.unaids.org/sites/default/files/country/documents/MWI_narrative_report_2015.pdf

[8] Kaida A, Laher F, Strathdee SA, Janssen PA, Money D, Hogg RS, et al Childbearing intentions of HIV-positive women of reproductive age in Soweto, South Africa: the influence of expanding access to HAART in an HIV hyperendemic setting. American journal of public health. 2011;101(2): 350–8. doi: 10.2105/AJPH.2009.177469 20403884 10.2105/AJPH.2009.177469 PMC3020203

[9] Schwartz SR, Mehta SH, Taha TE, Rees HV, Venter F, Black V. High pregnancy intentions and missed opportunities for patient-provider communication about fertility in a South African cohort of HIV-positive women on antiretroviral therapy. AIDS Behav. 2012;16(1): 69–78. doi: 10.1007/s10461-011-9981-3 21656145 10.1007/s10461-011-9981-3

[10] Melaku YA, Zeleke EG, Kinsman J, Abraha AK. Fertility desire among HIV-positive women in Tigray region, Ethiopia: implications for the provision of reproductive health and prevention of mother-to-child HIV transmission services. BMC Womens Health. 2014;14: 137 doi: 10.1186/s12905-014-0137-2 25407330 10.1186/s12905-014-0137-2 PMC4240867

[11] Sufa A, Wordofa MA, Wossen BA. Determinants of fertility intention among women living with HIV in western Ethiopia: implications for service delivery. Afr J Reprod Health. 2014;18(4): 54–60. 25854093

[12] Gutin SA, Namusoke F, Shade SB, Mirembe F. Fertility desires and intentions among HIV-positive women during the post-natal period in Uganda. Afr J Reprod Health. 2014;18(3): 67–77. 25438511

[13] Asfaw HM, Gashe FE. Fertility intentions among HIV positive women aged 18–49 years in Addis Ababa Ethiopia: a cross sectional study. Reprod Health. 2014;11: 36 doi: 10.1186/1742-4755-11-36 24885318 10.1186/1742-4755-11-36 PMC4038077

[14] Mmbaga EJ, Leyna GH, Ezekiel MJ, Kakoko DC. Fertility desire and intention of people living with HIV/AIDS in Tanzania: a call for restructuring care and treatment services. BMC Public Health. 2013;13: 86 doi: 10.1186/1471-2458-13-86 23360397 10.1186/1471-2458-13-86 PMC3577494

[15] Wagner G, Linnemayr S, Kityo C, Mugyenyi P. Factors associated with intention to conceive and its communication to providers among HIV clients in Uganda. Matern Child Health J. 2012;16(2): 510–8. doi: 10.1007/s10995-011-0761-5 21359828 10.1007/s10995-011-0761-5

[16] Beyeza-Kashesya J, Ekstrom AM, Kaharuza F, Mirembe F, Neema S, Kulane A. My partner wants a child: a cross-sectional study of the determinants of the desire for children among mutually disclosed sero-discordant couples receiving care in Uganda. BMC Public Health. 2010;10: 247 doi: 10.1186/1471-2458-10-247 20465794 10.1186/1471-2458-10-247 PMC2877675

[17] Haddad LB, Hoagland AB, Andes KL, Samala B, Feldacker C, Chikaphupha K, et al Influences in fertility decisions among HIV-infected individuals in Lilongwe, Malawi: a qualitative study. J Fam Plann Reprod Health Care 2017;43(3):210–2015. doi: 10.1136/jfprhc-2015-101395 27312425 10.1136/jfprhc-2015-101395

[18] Peltzer K, Chao LW, Dana P. Family planning among HIV positive and negative prevention of mother to child transmission (PMTCT) clients in a resource poor setting in South Africa. AIDS Behav. 2009;13(5): 973–9. doi: 10.1007/s10461-008-9365-5 18286365 10.1007/s10461-008-9365-5

[19] Mantell JE, Exner TM, Cooper D, Bai D, Leu CS, Hoffman S, et al Pregnancy intent among a sample of recently diagnosed HIV-positive women and men practicing unprotected sex in Cape Town, South Africa. Journal Acuir Immune Defic Syndr. 2014;67(4 Suppl): S202–9. 10.1097/QAI.0000000000000369 PMC4251915 25436819

[20] Nakayiwa S, Abang B, Packel L, Lifshay J, Purcell DW, King R, et al Desire for children and pregnancy risk behavior among HIV-infected men and women in Uganda. AIDS Behav. 2006;10(4 Suppl):S95–104. doi: 10.1007/s10461-006-9126-2 16715343 10.1007/s10461-006-9126-2

[21] National Statistical Office (NSO) and ICF Macro. 2011. *Malawi Demographic and Health Survey 2010* Zomba, Malawi, and Calverton, Maryland, USA: NSO and ICF Macro. https://dhsprogram.com/pubs/pdf/FR247/FR247.pdf

[22] Coutinho LM, Scazufca M, Menezes PR. Methods for estimating prevalence ratios in cross-sectional studies. Rev Saude Publica. 2008;42(6): 992–8. 19009156

[23] Kawale P, Mindry D, Phoya A, Jansen P, Hoffman RM. Provider attitudes about childbearing and knowledge of safer conception at two HIV clinics in Malawi. Reprod Health. 2015;12: 17 doi: 10.1186/s12978-015-0004-0 25771719 10.1186/s12978-015-0004-0 PMC4355153

[24] Matthews LT, Smit JA, Moore L, Milford C, Greener R, Mosery FN, et al Periconception HIV Risk Behavior Among Men and Women Reporting HIV-Serodiscordant Partners in KwaZulu-Natal, South Africa. AIDS Behav. 2015;19(12): 2291–303. doi: 10.1007/s10461-015-1050-x 26080688 10.1007/s10461-015-1050-x PMC4926315

[25] Litwin LE, Makumbi FE, Gray R, Wawer M, Kigozi G, Kagaayi J, et al Impact of Availability and Use of ART/PMTCT Services on Fertility Desires of Previously Pregnant Women in Rakai, Uganda: A Retrospective Cohort Study. Journal Acuir Immune Defic Syndr. 2015;69(3): 377–84. 10.1097/QAI.0000000000000612 PMC4506706 25835605

[26] Kaida A, Lima VD, Andia I, Kabakyenga J, Mbabazi P, Emenyonu N, et al The WHOMEN’s scale (Women’s HAART Optimism Monitoring and EvaluatioN Scale v.1) and the association with fertility intentions and sexual behaviours among HIV-positive women in Uganda. AIDS Behav. 2009;13(1 Suppl): 72–81. 19387819 10.1007/s10461-009-9553-y PMC3606958

[27] Harrington EK, Newmann SJ, Onono M, Schwartz KD, Bukusi EA, Cohen CR, et al Fertility intentions and interest in integrated family planning services among women living with HIV in Nyanza Province, Kenya: a qualitative study. Infect Dis in Obstet Gynecol. 2012;2012: 809682. 22844189 10.1155/2012/809682 PMC3403353

[28] Matthews LT, Beyez-Kashesya J, Cooke I, Davies N, Heffron R, Kaida A, et al Consensus statement: supporting safe conception and pregnancy for men and women living with and affected by HIV. AIDS Behav 2017 https://doi.org/10.1007/s10461-017-1777-7. 10.1007/s10461-017-1777-7 PMC5683943 28501964

[29] Davies NE, Matthews LT, Crankshaw TL, Cooper D, Schwartz SR. Supporting HIV prevention and reproductive goals in an HIV-endemic setting: taking safe conception services from policy to practice in South Africa. J Int AIDS Soc 2017;20(S1):21271. 28361506 10.7448/IAS.20.2.21271 PMC5577693

[30] Maier M, Andia I, Emenyonu N, Guzman D, Kaida A, Pepper L, et al Antiretroviral therapy is associated with increased fertility desire, but not pregnancy or live birth, among HIV+ women in an early HIV treatment program in rural Uganda. AIDS Behav. 2009;13 (1 Suppl): 28–37. 18389364 10.1007/s10461-008-9371-7 PMC3606959

[31] Iliyasu Z, Abubakar IS, Kabir M, Babashani M, Shuaib F, Aliyu MH. Correlates of fertility intentions among HIV/AIDS patients in northern Nigeria. Afr J Reprod Health. 2009;13(3): 71–83. 20690263

[32] Evens E, Tolley E, Headley J, McCarraher DR, Hartmann M, Mtimkulu VT, et al Identifying factors that influence pregnancy intentions: evidence from South Africa and Malawi. Cult Health Sex. 2015;17(3): 374–89. doi: 10.1080/13691058.2014.968806 25353696 10.1080/13691058.2014.968806

[33] Melaku Ya, Zeleke EG. Contraceptive utilization and associated factors among HIV positive women on chronic follow up care in Tigray Region, Northern Ethiopian: a cross-sectional study. PLOS ONE 2014;9(4):e94682 doi: 10.1371/journal.pone.0094682 24743241 10.1371/journal.pone.0094682 PMC3990566

[34] World Health Organization. Hormonal contraceptive eligibility for women at high risk of HIV 2016. http://apps.who.int/iris/bitstream/10665/254662/1/WHO-RHR-17.04-eng.pdf?ua=1. Accessed January 19, 2018.

[35] Scarsi KK, Darin KM, Nakalema D, Back DJ, Byakika-Kibwika P, Else LG, et al Unintended pregnancies observed with combined use of the levonorgestrel contraceptive implant and efavirez-based antiretroviral therapy: a three-arm pharmacokinetic evaluation of 48 weeks. Clin Infect Dis 2016;62(6):675–682. doi: 10.1093/cid/civ1001 26646680 10.1093/cid/civ1001 PMC4772838

[36] Morroni C, Bekker LG, Rees H. Contraceptive implants and efavirenz-based ART: friend or foe? Lancet HIV 2015;2(11):e454–5. doi: 10.1016/S2352-3018(15)00204-0 26520921 10.1016/S2352-3018(15)00204-0

