# Supplementary material for: Factors associated with desired fertility among HIV-positive women and men attending two urban clinics in Lilongwe, Malawi
Source: PLoS One. 2018 Jun 13;13(6):e0198798. doi: 10.1371/journal.pone.0198798 (PMC5999219; doi:10.1371/journal.pone.0198798)
Supplement: S1 Text — (DOCX) [file pone.0198798.s001.docx]

**Exploring the Impact of HIV and ART on Knowledge, Attitudes and Practices in Reproductive Health in Lilongwe, Malawi**

**QUANTITATIVE SURVEY**

**DATA ENTRY (SECTION Q)**

Q1. Enter the Participants ID Number: __________________________

Q2. Enter the Date (DD/MM/YYYY): ____________________________

Q3. Enter Research Assistant Name/Code: _______________________

Q4. Enter the survey start time: _________

**DEMOGRAPHICS (SECTION Z)**

Z1. Amuna kapena a akazi?

1. Akazi
2. Amuna

Z2. Kodi muli ndi zaka zingati?

Zaka_____ (Write “99” if “Don’t know”)

Z3. Kodi sukulu munalekeza pati?

1. Sindinaphunzire
2. Pulaimale sukulu
3. Ndinamaliza pulaimale
4. Ndinafikako ku sekondale
5. Ndinamaliza sekondale sukulu
6. Kuposa sekondale sukulu

Z4. Kodi mtundu wanu ndi chani?

1. Chewa
2. Lambya
3. Lomwe
4. Mang’anja
5. Ndali
6. Ngoni
7. Nkhonde
8. Sena
9. Tonga
10. Tumbuka
11. Yao
12. Zina ___________________________________________

Z5. Kodi ndinu a chipembedzo chanji?

1. Mipingo ina
2. Katolika
3. Musilamu
4. Wachikhalidwe
5. Opanda mpingo

Z6. Kodi panopo mumakhala kutauni kapena kumudzi? Kuti?

1. Kutauni
2. Kumudzi

Z7. Kodi mumatenga nthawi yaitali bwanji kuti mukafike ku kiliniki?

1. Mphindi zochepera 30
2. Pakati pa mphindi 30 ndi ola limodzi
3. Pakati pa ola limodzi ndi maola awiri
4. Pakati pa maola awiri ndi anayi
5. Kupitilira maola anayi

Z8.Ndi pafupifupi, maola angati pa mulungu amene mumagwira ntchito yolipidwa?

1. Palibe, sindigwira ntchito yolipidwa
2. Maola osakwana 10 pa mulungu
3. Pakati pa maola 10 ndi 20 pa mulungu
4. Pakati pa maola 20 ndi 40 pa mulungu
5. Maola oposa 40 pa mulungu

Z9.Kodi pansi pa nyumba yanu ndi pozila ndi chani?

1. Mtsiro/ dongo
2. Simenti
3. Zina (Tchulani): ___________________________

**HIV BACKGROUND (sECTION A)**

A1. Ndi chaka chiti chimene munapezeka ndi kachirombo ka HIV? (Perekani chaka chimene mukuganizira ngati simukukumbukira)

_______ *(Write “9999” if “Don’t Know”)*

A2. -> Calculate from Age in Question Z2: How old was participant when he/she first learned that he/she was HIV+? (Give an estimated age if unsure; if born with HIV, enter 0)

______ years old *(Write “99” if “Don’t Know”)*

**STIs & CONDOM USE (SECTION B)**

STIs

B1. Pa mwezi wathawu, kodi mumadandaula po zoti mwatenga matenda opatsirana pogonana?

1. Ayi
2. Inde

B2. Kodi munayamba mwapezekapo ndi matenda opatsirana pogonana pambali pa kachirombo ka HIV?

1. Ayi
2. Inde
3. Sindikudziwa

B3. Kodi munayamba mwapezekapo ndi matenda opatsirana pogonana (pambali pa kachirombo ka HIV) pa mwezi wathawu?

1. Ayi
2. Inde
3. Sindikudziwa

CONDOM USE

B4. Kodi munayamba mwagwiritsapo ntchito makondomu? *(If value=1(No), Skip to B8)*

1. Ayi
2. Inde

B5. Kodi munagwiritsapo ntchito makondomu pa mwezi wathawu?

1. Ayi
2. Inde

B6. Mwagwiritsa ntchito makondomu kangati?

1. Nthawi zina
2. Nthawi zonse

b6. Kodi munagwiritsa ntchito kondomu pa nthawi yomaliza imene munagonana?

1. Ayi
2. Inde

b7. N’chifukwa chiyani munagwiritsapo ntchito kondomu pa nthawi yomaliza imene munagonana? *(Sankhani yankho limodzi lokha)*

1. Kupewa mimba
2. Kupewa kutenga matenda opatsirana pogonana
3. Kupewa kupereka/kutenga ka chirombo ka HIV kwa ogonana nawo

B8. Kodi kugwiritsa ntchito kondomu kumathandiza kupewa kufala kwa kachirombo ka HIV?

1. Kumathandiza kwambiri
2. Kumathandiza pang’ono
3. Sikumathandiza

B9. Pa ubwenzi wanu, ndi ndani amene amayambitsa kuti mugwiritse ntchito kondomu?

- - - 1. Abambo
      2. Amayi
      3. Tnse tikhoza kuyambitsa

B10. Munayamba mwakhalapo pa ubwenzi pamene inu mumafuna kugwiritsa ntchito kondomu koma bwenzi lanu anakana kapena bwenzi lako linakuopsyeza? *(Monga kukuuza kuti athetsa chibwenzi)*

1. Ayi
2. Inde

**SEXUAL HISTORY AND CURRENT SEXUAL BEHAVIOR** **(SECTION C)**

C1. Kodi ndi ziti zimene zingafotokoze za ubwenzi wanu ndi amene mumagonana nawo? *(If value 3-5, Skip to C3)*

1. Banja
2. Pa ubwenzi wodalilika ndi munthu m’modzi yekha
3. Ndikuyenda ndi m’modzi kapena anthu ambiri
4. Sindilipabanja koma wosangalatsidwa kukhala ndi chibwenzi koma ndilibe pakali pano
5. Sindilipabanja ndipo sindikufuna chibwenzi

C2. Kodi mwakhala ndi bwenzi lanu kwa nthawi yaitali bwanji?

1. Miyezi itatu kapena kucheperapo
2. Pakati pa miyezi 3 ndi 6
3. Pakati pa miyezi 6 ndi chaka chimodzi
4. Pakati pa Chaka chimodzi ndi ziwiri
5. Pakati pa zaka ziwiri ndi zinayi
6. Kuposa zaka zinayi

C3. Kodi mungafotokoze za ubwenzi wanu ndi mzanu amene munagonana naye posachedwapa (amene munagwiritsa ntchito njira yoyamwana, njira ya abambo, kapena njira ya chimbuzi)?

1. Bwenzi lapabanja
2. Bwenzi la nthawi zonse
3. Maubwenzi ongosangalatsana
4. Wina woti munangokumana naye
5. Woyendayenda

6. Zina, tchulani ___________________

C4. Kodi mukuganiza kuti bwenzi lanu logonana nalo panopa limagonananso ndi anthu ena?

1. Ayi
2. Inde
3. Sindikudziwa

***READ: “Mafunso otsatirawa ndi wokhudza mchitidwe wogonana.***

***Ngati funso likuti ‘mtundu wina uli wonse wogonana’, likufunsa za wina uli onse ndi yonse ya zochitika izi: njira yoyamwana, njira ya abambo ndi njira ya chimbuzi. Nthawi ili yonse, funso lidzatsindika za mchitidwe wogonana umene ukunenedwawo. Ponena za bwenzi logonana nalo tikutanthauza munthu amene munagonana m’njira yoyamwana, njira ya abambo ndi njira ya chimbuzi.***

***Kwa mafunso amene akufunsa za maulendo, ngati simukukumbukira kuti ndi kangati, chonde perekani chiwerengero choyerekeza.”***

C5. Kodi munali ndi zaka zingati panthawi yoyamba pamene munagonanapo ndi munthu? *(Kugonana, tikutanthauza yoyamwana, njira ya abambo ndi njira ya chimbuzi)*

Zaka_______

C6. Kodi munali ndi zaka zingati panthawi yoyamba imene munagonana mnjira ya a abambo?

Zaka_______

C7. Kodi munakhalapo ndi abwenzi ogonana nawo angati chiyambire? Werengani wina ali yense amene munakhala naye, ngakhale iwo amene munagonana nawo m’njira ina kamodzi kokha. Ngati simukudziwa, perekani nambala yoyerekeza imene mungakwanitse.

1. Bwenzi m’modzi
2. Abwenzi awiri kufikira anayi
3. 5 kufikira10
4. 11 kufikira25
5. Abwenzi oposa 25

C8. Pamwezi wapitawo, munagonanapo ndi abwenzi angati? Werengani wina aliyense, ngakhale iwo amene munagonana nawo m’njira ina kamodzi kokha.

1. Bwenzi m’modzi
2. Abwenzi awiri kufikira anayi
3. 5 kufikira 10
4. 11 Kufikira 25
5. Abwenzi oposa 25
6. Sindinagonanepo ndi aliyense

C9. Pa mwezi wapitawu, munagonana mowirikiza bwanji mu njira ina ili yonse?

1. Sindinagone ndi munthu mwezi wathawu
2. Kamodzi mwezi wathawu
3. Kawiri mwezi wathawu
4. katatu kapena kanayi
5. kopose ra kanayi

C10. Mwa abwenzi amene munagonana nawo mwezi wathawu, mukuganiza kuti ndi angati abwenzi anu ogonana nawo amene akudziwa zoti muli ndi kachirombo ka HIV?

1. Palibe
2. Ena
3. Onse

C11. Mu mwezi wathawu, ndi angati mwa abwenzi anu ogonana nawo amene mumadziwa kuti ali ndi kachirombo ka HIV?

1. Palibe
2. Ena
3. Onse

C12. Kodi bwenzi lanu logonana nalo latsopano lili ndi kachirombo ka HIV?

1. Ayi
2. Inde
3. Sindikudziwa

C13. Kodi bwenzi latsopano likudziwa zoti inu muli ndi kachirombo ka HIV?

1. Ayi
2. Inde
3. Sindikudziwa

C14. Nthawi ina pa moyo wanu, alipo amene anakukakamizani kugonana kudzera mnjira yoyamwana, ya a abambo, kapena mnjira yachimbuzi inu musakufuna?

1. Ayi
2. Inde

C15. Nthawi ina pa moyo wanu, alipo amene anakuchitani nkhanza (kukumenyani, kukumenyani ndi faiti, kukumenyani mbama)?

1. Ayi
2. Inde

C16. Kodi munagonanapo posinthanitsa ndi ndalama, chakudya, mankhwala, kapena malo okhala?

1. Ayi
2. Inde

***READ: “Mafunso otsatirawa ndi okhudza kumwa mowa ndi kugwiritsa ntchito mankhwala ozunguza ubongo. Kumbukirani, mayankho anu onse ndi achinsinsi.”***

C17. Munayamba mwamwapo mowa? *(If value=1(No), then Skip to C19)*

1. Ayi
2. Inde

C18. Pa mwezi wapitawu, ndi pafupipafupi bwanji pamene mumamwa mowa musanagonane kapena panthawi yogonana?

1. Sindinapangepo
2. Nthawi zina (kamodzi kapena kambiri)
3. Nthawi zonse
4. Sindinagonanepo ndi munthu mwezi watha

C19. Munayamba mwagwiritsapo mankhwala ozunguza ubongo (Mwachitsanzo marijuana/chamba, khat)? *(If value=1(No), then Skip to DF1 if female, DM1 if male)*

1. Ayi
2. Inde

C20. Pa mwezi wapitawu, ndi pafupipafupi bwanji pamene mumagwirista ntchito mankhwala ozunguza bongo musanagonane kapena panthawi yogonana? *(If value=1(No), then Skip to DF1 if female, DM1 if male)*

1. Sindinapangepo
2. Nthawi zina
3. Nthawi
4. Sindinagonanepo ndi munthu mwezi watha

C21. Pa mwezi wapitawu, ndi mankhwala ati amene munagwiritsa ntchito musanagonana *(Sankhani zonse zimene zili zofunikira)*

1. Marijuana/chamba
2. Zina (Tchulani): ________

**FERTILITY PREFERENCES & PREGNANCY HISTORY (SECTION DF) (FEMALES ONLY)**

***READ: “Tsopano tifunsa mafunso okhudza mimba ndi mitu yokhudza mimba.”***

DF1.Kodi munayamba mwakhalapo ndi mimba? *(If value=1(No), Skip to DF15)*

1. Ayi
2. Inde

DF2. Kodi muli ndi ana angati?

1. Ndilibe ana
2. M’modzi
3. Awiri
4. Atatu
5. Anayi
6. Asanu kapena kupitilira apo

DF3. Kodi munali ndi zaka zingati pa nthawi yoyamba imene munatenga mimba?

zaka____

DF4. Kodi munayamba mwakhalapo ndi mtayo kapena kuberekapo mwana wakufa?

1. Ayi
2. Inde

DF5. Pa nthawi yomaliza imene munali ndi mimba, kodi munadziwa kuti muli ndi kachirombo ka HIV musanatenge mimba?

1. Ayi
2. Inde

DF6. Kodi munazindikira kuti muli ndi kachirombo ka HIV pa nthawi imene munali ndi mimba yanu yomaliza?

1. Ayi
2. Inde

DF7. Kodi munaberekapo mwana amene ali ndi kachirombo ka HIV?

1. Ayi
2. inde

DF8. Kodi alipo mwana wanu amene anamwalira ndi HIV/AIDs?

1. Ayi
2. Inde

DF9. Munayamba mwatengapo mimba yosakonzekera (mimba imene nthawi sinawerengedwe bwino, yosakonzekera, kapena yosafunika pa nthawi imene munatenga mimba)?

1. Ayi
2. Inde

DF10. Kodi ndi mimba zingati zosakonzekera zimene munakhala nazo?

Mimba________ zosakonzekera

DF11. Kodi munayamba mwachotsapo mimba mbuyomu (pa chifukwa china)? *(If value=1(No), Skip to DF15)*

1. Ayi
2. inde

DF12. Kodi munayamba mwachotsapo mimba chifukwa choti muli ndi HIV?

1. Ayi
2. Inde

DF13. Kodi bwenzi lanu linali ndi kachirombo ka HIV kapena ayi panthawi yomwe mumachotsa mimba?

1. Opanda kachirombo ka HIV
2. Wa kachirombo ka HIV
3. Ndamaliza mimba zoposa imodzi ndipo abwenzi anga ena anali ndi kachirombo ndinso ena opanda
4. Sindikudziwa

DF14. Kodi m’mene bwenzi lanu linalili pankhani ya kachirombo ka HIV zinakuthandizirani kuchotsa mimba?

1. Ayi
2. Inde

DF15. Pa miyezi 6 yapitayo, munali ndi nkhawa zotenga mimba?

1. Ayi
2. Inde

DF16. Kodi mukufuna kapena kukonzekera zokhala ndi ana ambiri (Nthawi ina ili yonse mtsogolomu)? *(If value-1(No), Skip to DF18)*

1. Ayi
2. Inde

DF17. Kodi mukufuna kudzakhala ndi mimba pa zaka ziwiri zikudzazi?

1. Ayi
2. Inde

DF18. Kodi mukukhulupilira kuti amayi amapanikizidwa kukhala ndi ana ngakhale kuti ali ndi ka chirombo ka HIV?

1. Ayi
2. Inde

DF19. Mutakhala kuti mwatenga mimba yosakonzekera kodi mungaganizire zochotsa?

1. Ayi
2. Inde
3. Sindikudziwa

DF20. Kodi kuchotsa mimba ndikoopsya kapena ayi?

1. Ayi
2. Inde
3. Sindikudziwa

DF21. Kodi kuuzidwa kuti muli ndi HIV zinasintha chifuniro chanu chofuna kapena kukonzekera kukhala ndi ana?

1. Ayi
2. Inde

DF22. Mukanakhala opanda HIV, kodi mukanakonda kukhala ndi ana ambiri?

1. Ayi
2. Inde

DF23. Pa miyezi 6 yapitayo, kodi munaganiza kuti simungathe kukhala ndi mimba chifukwa ndinu osabereka?

1. Ayi
2. Inde

DF24. Kodi bwenzi lanu logonana nalo latsopano likufuna ana ambiri?

1. Ayi
2. Inde
3. Panopo ndilibe bwenzi

DF25. Kodi mukuona kupanikizika kukhala ndi ana ambiri kuchokera kwa bwenzi lanu?

1. Ayi
2. Inde

DF26. Kodi mukuona kupanikizika kukhala ndi ana ambiri kuchokera kwa banja lanu?

1. Ayi
2. Inde

DF27. Kodi mukuona kupanikizika kukhala ndi ana ambiri kuchokera ku dera lanu?

1. Ayi
2. Inde

DF28. Kodi mukuona ngati kutenga mimba kutha kupangitsa moyo wanu kukhala wanthazi kapena ayi

1. Wanthanzi

2. Wosakhala wathanzi

DF29. Mumaganizo anu, kodi mukuganiza kuti anthu ambiri sagwirizana nazo zoti amayi akachirombo akhale ndi ana?

1. Ayi
2. Inde

DF30. Mumaganizo anu, kodi ndi ufulu wa aliyense kukhala ndi mwana, posawerengera za kachirombo ka HIV?

1. Ayi
2. Inde

DF31. Kodi amayi amene ali ndi kachirombo ka HIV angathe kubereka ana opanda kachirombo?

1. Ayi
2. Inde

DF32. Kodi mukudziwa ngati mankhwala alipo othandiza kuteteza kachiromboka HIV kuchoka kwa mayi kupita kwa mwana?

1. Ayi
2. Inde

***READ: “Tsopano tikufuna kudziwa za zokambirana zanu ndi adokotala kapena anthu ena ogwira ntchito zokhudza kulera ndi kugonana. Kumbukirani, mayankho anu sitidzagawana ndi ena mwa amene adzakupatseni chithandizo. Tikufuna kudziwa za m’mene mukumvera moona mtima zokhudza zokambirana zanu za kulera ndi amene amakupatsani chisamaliro cha HIV.”***

DF33. Kodi ogwira ntchito kuchipatala amakugwetsani mphwayi kuti mubereke mutadziwa za m’mene mulili pa nkhani ya kachirombo ka HIV?

1. Ayi
2. Inde

DF34. Kodi mukuganiza kuti mungakhale ndi chilimbikitso kuchokera kwa dokotala mutasankha kukhala ndi ana ambiri?

1. Ayi
2. Inde

DF35. Kodi mukuganiza kuti dokotala wanu ndi/ kapena wogwira ntchito za umoyo akupatsani uthenga wokwanira kuti mupange chiganizo choyenera pa njira yoyenera ya kulerakuti inu mugwiritse ntchito?

1. Ayi
2. Inde

DF36.Kodi munayamba mwakhalapo ndi zokambirana ngati banja zokhudza chinyontho kapena kulera?

- - - 1. Ayi
      2. Inde

DF37. Kodi mukuganiza kuti ndi zothandiza ngati mabanja akukumana pamodzi ndi opereka chisamaliro kapena phungu kukambirana za kulera?

1. Ayi
2. Inde

DF38. Kodi mungathe kukhala omasuka kumva ndi kulankhula za kulera pagulu?

1. Ayi
2. Inde

**FERTILITY PREFERENCES & PREGNANCY HISTORY (SECTION DM) (MALES ONLY)**

***READ: “Tsopano tifunsa zokhudza mimba ndi mitu yokhudza mimba.”***

DM1. Kodi munapelekako mimba? *(If value=1(No), Skip to DM15)*

1. Ayi
2. Inde

DM2. Kodi muli ndi ana angati?

1. Ndilibe ana
2. M’modzi
3. Awiri
4. atatu
5. Anayi
6. Asanu kapena kuposa apo

DM3. Kodi munali ndi zaka zingati pamene bwenzi lanu linatenga mimba?

zaka____

DM4. Nthawi yomaliza imene bwenzi lanu linatenga mimba, kodi munadziwa kuti munali ndi kachirombo ka HIV?

1. Ayi
2. Inde

DM5. Kodi munakhalapo ndi mwana amene anabadwa ndi kachirombo ka HIV?

1. Ayi
2. Inde

DM6. Kodi mwana wanu wina anamwalirapo ndi HIV/AIDs?

1. Ayi
2. Inde

DM7. Kodi bwenzi lanu linayamba latengapo mimba yosakonzekera (mimba imene inabwera pa nthawi yolakwika, yosakonzekera, kapena yosafunika pa nthawi imene mimba inabwera)?

1. Ayi
2. Inde

DM8. Pa miyezi 6 yapitayo, kodi munaderapo nkhawa zoti bwenzi lanu latenga mimba?

1. Ayi
2. Inde

DM9. Kodi mumafuna kapena kukonzekera kukhala ndi ana ambiri (pa nthawi ina mtsogolo)? *(If value-1(No), Skip to DM11)*

1. Ayi
2. Inde

DM10. Kodi mukufuna kuti bwezi lanu lidzatengemimba zaka ziwiri zikubweraz?

1. Ayi
2. Inde

DM11. Kodi mumakhulupilira kuti amayi amaumilizika kukhala ndi ana, ngakhale mayi ali ndi kachirombo ka HIV?

1. Ayi
2. Inde

DM12. Kodi bwenzi lanu litatenga mimba yosakonzekera, kodi mungaganizire zokachotsa?

1. Ayi
2. Inde
3. Sindikudziwa

DM13. Kodi mumakhulupilira kuti kuchotsa mimba ndi kwabwino?

1. Ayi
2. Inde
3. Sindikudziwa

DM14. Kodi kuuzidwa kuti muli ndi kachirombo ka HIV kumasintha chifuniro chanu kapena chiyembekezo chanu chokhala ndi ana?

1. Ayi
2. Inde

DM15 Mukanakhala kuti mulibe kachirombo ka HIV, kodi mukadafuna kukhala ndi ana ambiri?

1. Ayi
2. Inde

DM16. Kodi bwenzi lanu latsopano likufuna ana ambiri?

1. Ayi
2. Inde
3. Pakali pano ndilibe bwenzi

DM17. Kodi mukuona kupanikizika kukhala ndi ana ambiri kuchokera kwa bwenzi lanu?

1. Ayi
2. Inde

DM18. Kodi mukuona kupanikizika kukhala ndi ana ambiri kuchokera kwa a banja lanu?

1. Ayi
2. Inde

DM19. Kodi mukuona kupanikizika kukhala ndi ana ambiri kuchokera kwa anthu a kudera lanu?

1. Ayi
2. Inde

DM20. Mumaganizo anu, kodi mukuganiza kuti anthu ambiri sagwirizana nazo zoti amayi akachirombo akhale ndi ana?

1. Ayi
2. Inde

DM21. Mumaganizo anu, kodi ndi ufulu wa wina ali yense kukhala ndi mwana, posawerengera zoti ali ndi kachirombo ka HIV?

1. Ayi
2. Inde

DM22. Kodi amayi amene ali ndi kachirombo ka HIV angathe kubereka ana opanda kachirombo?

1. Ayi
2. Inde

DM23. Kodi mukudziwa ngati mankhwala akupezeka oteteza kachiromboka HIV kuchoka kwa mayi kupita kwa mwana?

1. Ayi
2. Inde

DM24. Kodi mukuganiza kuti adokotala anu / ogwira ntchito za umoyo akukupatsani uthenga wokwanira kuti mupange chiganizo choyenera pa njira yogwiritsa ntchito ya kulera?

1. Ayi
2. Inde

DM25. Kodi munayamba mwakhalapo ndi zokambirana ngati banja zokhudza chinyontho kapena kulera?

1. Ayi
2. Inde

DM26. Kodi mukuganiza kuti ndizofunikira ngati mabanja akukumana pamodzi ndi opereka chisamaliro kapena phungu kukambirana za kulera?

1. Ayi
2. Inde

DM27. Kodi mungathe kukhala omasuka kumva ndi kulankhula zokhudza kulera pagulu?

1. Ayi
2. Inde

**CONTRACEPTIVE KNOWLEDGE/ATTITUDES (sECTION EF) (FEMALES ONLY)**

**READ: “*Ndi mafunso otsatirawa tiona mlingo wa nzeru zimene muli nazo ndi zimene simumamvetsetsa zokhudza njira za kulera.”***

EF1. Kodi munamvapo za njira zina za kulera zimene zingathe kubweretsa mavuto pa munthu amene ali ndi kachirombo ka HIV(Kuwonjezera mavuto a HIV)? *(If value = 1(No), Skip to EF3)*

1. Ayi
2. Inde

EF2. Ndi njira iti ya kulera (ziti) imene munamvapo kuti imabweretsa zovuta pa anthu omwe ali ndi kachirombo ka HIV (Kuwonjezera mavuto a HIV)? (*Sankhani zonse zimene zili zoyenera)*

1. Makondomu
2. Jakisoni
3. Mapilisi
4. Lupu
5. Jadere
6. Palibe

Ef3. Ndi njira iti yakulera imene munamva kuti musagwiritse ntchito pamene mukumwa mankhwala a HIV? Sankhani zonse zimene zili zoyenera*.*

1. Makondomu
2. Jakisoni
3. Mapilisi
4. Lupu
5. Jadere
6. Palibe

EF4. Kodi munamvapo kuti njira za kulera zimapangitsa kukhala kovuta kutenga mimba mtsogolo? *(If value=1(No), Skip to EF6)*

1. Ayi
2. Inde

EF5. Ndi njira iti ya kulera, imene munamva kuti imapangitsa kukhala kovuta kutenga mimba mtsogolo? *(*Sankhani zonse zimene zili zoyenera*)*

1. Makondomu
2. Jakisoni
3. Mapilisi
4. Lupu (IUCD)
5. Jadelle
6. Palibe

EF6. Lisanafike lero, munali mutamvapo za lupu? *(If value=1(No), Skip to EF8)*

1. Ayi
2. Inde

EF7. Lisanafike lero, munali mutamvapo za njira kulera ya implant, imene imatchedwanso kuti Jadelle, SinoImplant kapena Norplant?

1. Ayi
2. Inde

EF8. Kodi kumanga chingwe mchiuno mwa amayi (mwa chitsanzo ndi mzamba) kumateteza kutenga mimba?

1. Ayi
2. Inde

EF9. Kodi mukuganiza kuti makondomu ndi othandiza poteteza mimba?

1. Ayi
2. Inde

EF10. Ngati mukugwiritsa ntchito njira ya kulera, kodi mukuganiza kuti ndibwino kugwiritsanso kondomu?

1. Ayi
2. Inde

EF11. Kodi mafuta amukondomu amatupitsa mimba ya amayi?

1. Ayi
2. Inde
3. Sindikudziwa

EF12. Kodi majakisoni ndi mapilisi zimasokoneza chilakolako chofuna kugonana kwa mayi?

1. Jakisoni kapena mapilisi ssasokoneza chikolako cha mayi chofuna kugonana
2. Mapilisi okha amsokoneza chilakolako cha mayi
3. Jakisoni yekha amasokoneza chilakolako cha mayi
4. Jakisoni ndi ma pilisi amasokoneza chilakolako cha mayi
5. Sindikudziwa

EF13. Kodi mayi amene akumwa mapilisi kapena kugwiritsa ntchito jakisoni amasokoneza chilakolako chamwamunaamene akufuna kugona naye?

1. Jakisoni kapena mapilisi ssasokoneza chikolako cha abambo chofuna kugonana
2. Mapilisi okha amsokoneza chilakolako cha abambo
3. Jakisoni yekha amasokoneza chilakolako cha abambo
4. Jakisoni ndi ma pilisi amasokoneza chilakolako cha abambo
5. Sindikudziwa

EF14. Kodimukuganiza kuti njira za kulera zomwe zimakhala ndi michere yamthupi imayambitsa cancer?

1. Ayi
2. Inde
3. Sindikudziwa

EF15. ndi njira iti ya kulera imene imateteza kupatsira kachirombo ka HIV kwa bwenzi lanu? *(Sankhani zonse zimene zili zoyenera)*

1. Jakisoni
2. Mapilisi
3. Lupu
4. Jadelle
5. Makondomu
6. Kuchotsa
7. Njira za pangozi
8. Kudziletsa
9. Kukhala ndi bwenzi limodzi
10. Palibe pa njira zonsezi

EF16. Ndi njira iti, ziti zakulera zimene zimachulutsa mpata wopereka kachirombo ka HIV kwa bwenzi lanu? (Sankhani zonse zimene zili zoyenera)

1. Jakisoni
2. Mapilisi
3. Lupu
4. Jadelle
5. Makondomu
6. Kuchotsa
7. Njira za pangozi
8. Kudziletsa
9. Kukhala ndi bwenzi limodzi
10. Palibe pa njira zonsezi

EF17. Kodi mumaphunzira kuti zokhudza kulera? *(Sankhani zonse zimene zili zoyenera)*

1. Kuchokera kwa a m’banja
2. Kuchokera kwa abwenzi ndi/ kapena oyandikana nawo
3. Kuchokera kwa ogwira ntchitoza umoyo m’midzi
4. Kuchokera kwa namwino kukiliniki
5. Kwa dokotala ku kiliniki
6. Kuchokera ku bungwe la Family Planning Association of Malawi (FPAM)
7. Kuchokera kwa azamba
8. Kuchokera pa wailesi kapena pa kanema
9. Kuchokera mumanyuzipepala kapena ma magazini
10. Kuchokera mu zinthu zina, chonde tchulani __________

EF18. Ndi kuti kumene mungafune kukalandilira uthenga wokhudza njira zakulera?

1. Kunyumba
2. Ku kiliniki
3. Msokhano wa m’mudzi
4. Radio
5. Zina, chonde tchulani ______________

EF19. Ndi kuti kumene mungafune kukalandilira njira za kulera?

1. Kunyumba
2. Ku kiliniki
3. Ku chipatala chaching’ono
4. Ku pharmacy
5. Zina, tchulani

**CONTRACEPTIVE KNOWLEDGE/ATTITUDES (sECTION EM) (MALES ONLY)**

***READ: “Ndi mafunso otsatirawa tikufuna kuona mlingo wa zomwe mukuziwa ndi zimene simumvetsetsa zokhudza njira za kulera.”***

EM1. Kodi munamvapo zoti njira za kulera zimapangitsa kuti kukhale kovuta kutenga mimba mtsogolo? *(If value=1(No), Skip to EM3)*

1. Ayi
2. Inde

EM2. Ndi njira iti/ziti za kulera zimene munamvapo kuti zimapangitsa kukhala kovuta kutenga mimba mtsogolo? *(Sankhani zonse zimene zili zoyenera)*

1. makondomu
2. Jakisoni
3. Mapilisi
4. Lupu
5. Jadelle
6. Palibe pa njira zonsezi

EM3. Kodi kumanga chingwe mchiuno mwa amayi (mwa chitsanzo ndi mzamba) kumateteza kutenga mimba?

1. Ayi
2. Inde

EM4. Kodi mukuganiza kuti makondomu ndi othandiza poteteza mimba?

1. Ayi
2. Inde

EM5. Ngati bwenzi lanu likugwiritsa ntchito njira ya kulera, kodi mukuganiza kuti ndibwino kuti inu mugwiritsenso ntchito kondomu?

1. Ayi
2. Inde

EM6. Kodi majakisoni ndi mapilisi zimasokoneza chilakolako chofuna kugonana kwa mayi?

1. Jakisoni kapena mapilisi ssasokoneza chikolako cha mayi chofuna kugonana
2. Mapilisi okha amsokoneza chilakolako cha mayi
3. Jakisoni yekha amasokoneza chilakolako cha mayi
4. 4.jakisoni ndi ma pilisi amasokoneza chilakolako cha mayi
5. Sindikudziwa

EM7. Kodi mayi amene akumwa mapilisi kapena kugwiritsa ntchito jakisoni amasokoneza chilakolako chofuna kugonana cha mwamuna amene akufuna kugona naye?

1. Jakisoni kapena mapilisi ssasokoneza chikolako cha abambo chofuna kugonana
2. Mapilisi okha amsokoneza chilakolako cha abambo
3. Jakisoni yekha amasokoneza chilakolako cha abambo
4. Jakisoni ndi ma pilisi amasokoneza chilakolako cha abambo
5. Sindikudziwa

EM8. Kodi mumakhulupilira kuti njira za kulera zimene zimakhala ndi michere ya nthupi zimayambitsa matenda a cancer?

1. Ayi
2. Inde
3. Sindikudziwa

EM9. ndi njira iti ya kulera imene imateteza kupatsira kachirombo ka HIV kwa bwenzi lanu? *(Sankhani zonse zimene zili zoyenera)*

1. Jakisoni
2. Mapilisi
3. Lupu
4. Jadelle
5. Makondomu
6. Kuchotsa
7. Njira za pangozi
8. Kudziletsa
9. Kukhala ndi bwenzi limodzi
10. Palibe pa njira zonsezi

EM10. Ndi njira ziti zakulera zimene zimachulutsa mpata wopereka kachirombo ka HIV kwa bwenzi lanu? *(Sankhani zonse zimene zili zoyenera)*

1. Jakisoni
2. Mapilisi
3. Lupu
4. Jadelle
5. Makondomu
6. Kuchotsa
7. Njira za pangozi
8. Kudziletsa
9. Kukhala ndi bwenzi limodzi
10. Palibe pa njira zonsezi

**FEMALE CONTRACEPTIVE USE (SECTION FF)** **(FEMALES ONLY)**

*This section is for women only; men should respond to contraceptive use questions in Section FM instead.*

***READ: “Tifunsa za mtundu wa njira za kulera (zotchedwanso ma contraceptives), ngati ina, imene munagwiritsapo ntchito ndi zimene munakumana nazo pogwiritsa ntchito.”***

FF1. Kodi munayamba mwagwiritsapo ntchito njira ya kulera? (Ponena kuti njira ya kulera tikutanthauza kugwiritsa ntchito njira yoteteza kutenga mimba monga mapilisi, lupu, kutseka kwa a mayi kapena abambo, etc.) *(If value = 1(No), Skip to G1)*

1. Ayi
2. Inde

FF2. Kodi mukugwiritsa njira ina ili yonse yakulera pakali pano?

1. Ayi
2. Inde

FF3. Kodi mumagwiritsa ntchito njira ina ili yonse ya kulera (pambali pa makondomu) pa nthawi yomaliza imene munagonana? (*If value=1(No), then Skip to FF5)*

1. Ayi
2. Inde

FF4. Ndi njira iti ya kulera imene munagwiritsa ntchito pa nthawi yomaliza imene munagonana? *(Sankhani zonse zimene zili zoyenera)*

1. Makondomu
2. Lupu(IUCD)
3. Jadelle
4. Jakisoni (Depo Provera)
5. Mapilisi
6. Kutaila kunja umuna
7. Njira yakulera ya pangozi
8. Kutseka kwa amayi/ kumanga thumbo
9. Kutseka kwa abambo/ vasectomy
10. Zina, tchulani: _____________________________________

FF5. Kodi chipembedzo chanu chimathandizira kupanga chiganizo chofuna kugwiritsa ntchito njira ya kulera kapena ayi?

1. Ayi
2. Inde

FF6. Kodi pali mankhwala amene mukumwa amene akuthandizira kupanga chiganizochofuna kugwiritsa ntchito njira ya kulera kapena ayi?

1. Ayi
2. Inde
3. Sizoyenera (Sindili pa mankhwala ena ali onse)

ORAL CONTRACEPTIVES

F7. Kodi munayamba mwagwiritsapo ntchito njira ya mapilisi? *(If value = 1(No), Skip to FF12 [Depo provera])*

1. Ayi
2. Inde

FF8. Kodi ndi nthawi yaitali bwanji imene munakhala mukugwiritsa ntchito mapilisi?

1. Kuchepera mwezi umodzi
2. Mwezi umodzi kufikira itatu
3. Mwezi inayi kufikira isanu ndi umodzi
4. Miyezi isanu ndi iwiri kufikira chaka chimodzi
5. Kuposa chaka chimodzi

FF9. Kodi munagwiritsapo ntchito mapilisi mwezi wapitawo?

1. Ayi
2. Inde

FF10. Kodi munali wokhutitsidwa bwanji ndi kugwiritsa ntchito mapilisi?

1. Wokhutitsidwa kwambiri
2. Wokhutitsidwa nthawi zambiri
3. Wosakhutitsidwa nthawi zambiri
4. Wosakhutitsidwa nkomwe

FF11. Mutagwiritsa ntchito mapilisi, kodi munakumana ndi zina mwa zotsatirazi? *(Sankhani zonse zimene zili zoyenera)*

1. Msambo unawonjeza
2. Msambo unakhala bwino
3. Msambo unasiya
4. Mutu kupweteka
5. Sikero kukwera
6. Kuthothoka kwa tsitsi
7. Kusalala kwa khungu
8. Sindinali ndi zina mwa zimenezi

DEPO PROVERA

FF12. Kodi munayamba mwagwiritsapo ntchito njira ya jakisoni? *(If value = 1(No), Skip to F18 [Implant])*

1. Ayi
2. Inde

FF13. Kodi ndi nthawi yaitali bwanji imene munakhala mukugwiritsa ntchito jakisoni?

1. Kuchepera mwezi umodzi
2. Mwezi umodzi kufikira itatu
3. Miyezi inayi kufikira isanu ndi umodzi
4. Miyezi isanu ndi iwiri kufikira chaka chimodzi
5. Kuposa chaka chimodzi

FF14. Kodi munalandira jakisoni wanu omaliza pa nthawi yoyenerera?

1. Ayi
2. Inde
3. Anali jakisoni wanga woyamba

FF15. Kodi munagwiritsapo ntchito jakisoni mwezi wapitawo?

1. Ayi
2. Inde

FF16. Kodi munali wokhutitsidwa bwanji ndi kugwiritsa ntchito jakisoni?

1. Wokhutitsidwa kwambiri
2. Wokhutitsidwa nthawi zambiri
3. Wosakhutitsidwa nthawi zambiri
4. Wosakhutitsidwa nkomwe

FF17. Mutagwiritsa ntchito jakisoni, kodi munakumana ndi zina mwa zotsatirazi? *(Sankhani zonse zimene zili zoyenera)*

1. Msambo unawonjeza
2. Msambo unakhala bwino
3. Msambo unasiya
4. Mutu kupweteka
5. Sikero kukwera
6. Kuthothoka kwa tsitsi
7. Kusalala kwa khungu
8. Sindinali ndi zina mwa zimenezi
9. Sikero inatsika

IMPLANT

FF18. Kodi munayamba mwagwiritsapo ntchito njira ya jadelle (norplant, jadelle or sinoimplant)? *(If value = 1(No), Skip to FF23 [IUCD])*

1. Ayi
2. Inde

FF19. Kodi ndi nthawi yaitali bwanji imene munakhala mukugwiritsa ntchito jadelle?

1. Kuchepera mwezi umodzi
2. Mwezi umodzi kufikira itatu
3. Mwezi inayi kufikira isanu ndi umodzi
4. Miyezi isanu ndi iwiri kufikira chaka chimodzi
5. Kuposa chaka chimodzi

FF20. Kodi munagwiritsapo ntchito jadelle mwezi wapitawo?

1. Ayi
2. Inde

FF21. Kodi munali wokhutitsidwa bwanji ndi kugwiritsa ntchito jadelle?

1. Wokhutitsidwa kwambiri
2. Wokhutitsidwa nthawi zambiri
3. Wosakhutitsidwa nthawi zambiri
4. Wosakhutitsidwa nkomwe

FF22. Mutagwiritsa ntchito jadelle, kodi munakumana ndi zina mwa zotsatirazi? *(Sankhani zonse zimene zili zoyenera)*

1. Msambo unawonjeza
2. Msambo unakhala bwino
3. Msambo unasiya
4. Mutu kupweteka
5. Kukwera sikero
6. Kuthothoka kwa tsitsi
7. Kutsika sikero
8. Kusalala kwa khungu
9. Sindinali ndi zina mwa zimenezi

IUCD

FF23. Kodi munayamba mwagwiritsapo ntchito njira ya lupu? *(If value = 1(No), Skip to FF28 [Other])*

1. Ayi
2. Inde

FF24. Kodi ndi nthawi yaitali bwanji imene munakhala mukugwiritsa ntchito lupu?

1. Kuchepera mwezi umodzi
2. Mwezi Umodzi kufikira itatu
3. Mwezi inayi kufikira isanu ndi umodzi
4. Miyezi isanu ndi iwiri kufikira chaka chimodzi
5. Kuposa chaka chimodzi

FF25. Kodi munagwiritsapo ntchito lupu mwezi wapitawo?

1. Ayi
2. Inde

FF26. Kodi munali wokhutitsidwa bwanji ndi kugwiritsa ntchito lupu?

1. Wokhutitsidwa kwambiri
2. Wokhutitsidwa nthawi zambiri
3. Wosakhutitsidwa nthawi zambiri
4. Wosakhutitsidwa nkomwe

FF27. Mutagwiritsa ntchito lupu, kodi munakumana ndi zina mwa zotsatirazi? *(Sankhani zonse zimene zili zoyenera)*

1. Msambo unawonjeza
2. Msambo unakhala bwino
3. Msambo unasiya
4. Mutu kupweteka
5. Kukwera sikero
6. Kuthothoka kwa tsitsi
7. Kutsika sikero
8. Kusalala kwa khungu
9. Sindinali ndi zina mwa zimenezi

OTHER

FF28. Kodi mukudziwapo za njira ya kulera imene ingagwiritsidwe ntchito mutagonana mosadziteteza poteteza kutenga mimba? *(If value=1, Skip to FF32)*

1. Ayi
2. Inde

FF29. Mwa njira zotsatirazi ndi ziti zimene munamvapo kuti zimateteza mimba zikagwiritsidwa ntchito mutatha kugonana? *(Sankhani zonse zimene zili zoyenera)*

- - - 1. Kutawasa
      2. Kuika zitsamba m’njira ya abambo
      3. Mapilisi a kulera
      4. Lupu
      5. Kutayira umuna pansi
      6. Kondomu
      7. Jakisoni
      8. Jadelle
      9. Zina:___________________________

FF30. Kodi munayamba mwagwiritsapo ntchito imodzi mwa njirazi mutatha kugonana pofuna kuteteza mimba?

1. Ayi *(Skip to FF32*)
2. Inde

FF31. Ndi njira iti imene munayamba mwagwiritsirapo ntchito mutatha kugonana? *(Sankhani zonse zimene zili zoyenera)*

1. Kutawasa
2. Kuika zitsamba m’njira ya abambo
3. Mapilisi a kulera
4. Lupu
5. Kutayira umuna pansi
6. Kondomu
7. Jackisoni
8. Jadelle
9. Zina: ____________________________

FF32. Kodi munayamba mwagwiritsapo ntchito zina mwa njira zakulera zotsatirazi? *(Sankhani zonse zimene zili zoyenera. If value=7 (none of the above), Skip to FF34)*

1. Njira yotchinga khomo la chiberekero (Diaphragm)
2. Njira yopha umuna
3. Yochotsa mimba
4. Yotaila kunja umuna
5. Yotseka abambo
6. Yotseka amayi
7. Sindinagwiritsepo ina mwa njirazi

FF33. Kodi munayamba mwagwiritsapo ntchito ina mwa njirazi pa mwezi wapitawu? *(Sankhani zonse zimene zili zoyenera)*

1. Njira yotchinga khomo la chiberekero (Diaphragm)
2. Njira yopha umuna
3. Yochotsa mimba
4. Kutaila kunja umuna
5. Yotseka abambo
6. Yotseka amayi
7. Sindinagwiritsepo ina mwa njirazi

***READ: “Mafunso otsatirawa ndi okhudza amene munamuuza pamene mumapanga nchito chiganizo chogwiritsa ntchito njira yakulera imene mukugwiritsa ntchito pakali pano.”***

FF34. Mene mumaganizira njira ya kulera yomwe mukugwiritsa ntchito pakadali pano kodi munakambilanako ndi okondedwa wanu za njira yoti mugwiritse ntchito?

1. Inde
2. Ayi

FF35. Mene mumaganizira njira ya kulera yomwe mukugwiritsa nchito pakadali pano kodi munakambilanako ndi alangizi azaumoyo apa chipatala panu za njira yoti mugwiritse ntchito?

1. Inde
2. Ayi

FF36. Mene mumaganizira njira ya kulera yomwe mukugwiritsa ntchito pakadali pano kodi munakambilanako ndi aphunzitsi a mudzi pa za njira yoti mugwiritse ntchito?

1. Inde
2. Ayi

FF37. Mene mumaganizira njira ya kulera yomwe mukugwiritsira ntchito pakadali pano kodi munakambilanako ndi achibale anu kapena anzanu pa za njira yoti mugwiritse ntchito?

1. Inde
2. Ayi

FF38. Mmene mumaganizira njira ya kulera yomwe mukugwiritsa ntchito padakali pano kodi munapeza uthenga umenewu kuchokera ku magazini, kanema, internet, sukulu, mabuku, kapena radio pa njira yoti mugwiritse ntchito?

1. Magazini
2. Kanema
3. Internet
4. Sukulu
5. Mabuku
6. Radio
7. Zina: ___________________________________

****Skip to G1 after completing this section.***

**MALE CONTRACEPTIVE USE (SECTION FM) (FOR MALES ONLY)**

***READ: “Tsopano tifunsa za mafunso okhudza mitundu ya kulera (yotchedwanso contraceptives), ngati ilipo, imene bwenzi/abwenzi anu anagwiritsapo ntchito ndi zimene munakumana nazo zokhudza njirazi.”***

FM1. Kodi inu kapena wina mwa abwenzi anu anayamba wagwiritsapo ntchito njira za kulera? (Ponena kuti kulera tikutanthauza kugwiritsa ntchito chinthu china pofuna kuteteza mimba monga kugwiritsa ntchito makondomu, mapilisi, jakisoni, kapena kutseka kwa amayi/kutseka kwa abambo.) *(If value = 1(No), Skip to G1])*

1. Ayi
2. Inde
3. Sindikudziwa

FM2. Kodi inu kapena bwenzi lanu likugwiritsa ntchito njira ina ili yonse yakulera pakali pano?

1. Ayi
2. Inde

FM3. Kodi inu kapena bwenzi lanu likugwiritsa ntchito njira ina ili yonse yakulera pa nthawi yomaliza imene munagonana? *(If value=1(No), then Skip to FM5)*

1. Ayi
2. Inde

FM4. Ndi njira iti ya kulera imene inu ndi bwenzi lanu limagwiritsa ntchito pa nthawi yomaliza imene munagonana? *(Sankhani zonse zimene zili zoyenera)*

1. Kondomu
2. Lupu
3. Jadelle
4. Jakisoni (Depo Provera)
5. Mapilisi
6. Yotaila kunja umuna
7. Njira yakulera ya pangozi
8. Kutseka kwa abambo/ vasectomy
9. Kutseka kwa amayi/ kumanga thumbo
10. Zina, tchulani: _________________

FM5. Kodi chipembedzo chanu chimathandizira kuti inu kapena bwenzi lanu mupange chiganizo chofuna kugwiritsa ntchito njira ya kulera kapena ayi?

1. Ayi
2. Inde

OCPs

FM6. Kodi abwenzi anu anayamba agwiritsapo ntchito mapilisi? *(If value = 1(No), Skip to FM9 [Depo provera])*

1. Ayi
2. Inde
3. Sizoyenera

FM7. Kodi alipo wina mwa abwezi anu amene anagwiritsa ntchito mapilisi mwezi wapitawu?

- - - 1. Ayi
      2. Inde
      3. Sindikudziwa
      4. Sindinakhalepo ndi bwenzi pa mwezi wathawu

FM8. Kodi munali wokhutitsidwa bwanji inu ndi bwenzi lanu pogwiritsa ntchito mapilisi?

1. Wokhutitsidwa kwambiri
2. Wokhutitsidwa nthawi zambiri
3. Wosakhutitsidwa nthawi zambiri
4. Wosakhutitsidwa nkomwe

DEPO PROVERA

FM9. Kodi alipo mwa abwenzi anu amene anagwiritsapo ntchito jakisoni? *(If value = 1(No), Skip to FM12 [Implant])*

1. Ayi
2. Inde
3. Sindikudziwa

FM10. Kodi bwezi lanu linagwiritsapo ntchito jakisoni mwezi wapitawu?

1. Ayi
2. Inde
3. Sindikudziwa
4. Sindinakhalepo ndi bwenzi pa mwezi wathawu

FM11. Kodi munali wokhutitsidwa bwanji inu ndi bwenzi lanu pogwiritsa ntchito jakisoni?

1. Wokhutitsidwa kwambiri
2. Wokhutitsidwa nthawi zambiri
3. Wosakhutitsidwa nthawi zambiri
4. Wosakhutitsidwa nkomwe

IMPLANT

FM12. Kodi alipo mwa abwenzi anu amene anagwiritsapo ntchito jadelle? (Norplant, jadelle or sinoimplant)? *(If value = 1(No), Skip to FM15 [IUCD])*

1. Ayi
2. Inde
3. Sindikudziwa

FM13. Kodi bwezi lanu linagwiritsapo ntchito jadelle mwezi wapitawu?

1. Ayi
2. Inde
3. Sindikudziwa
4. Sindinakhalepo ndi bwenzi pa mwezi wathawu

FM14. Kodi munali wokhutitsidwa bwanji inu ndi bwenzi lanu pogwiritsa ntchito jadelle?

1. Wokhutitsidwa kwambiri
2. Wokhutitsidwa nthawi zambiri
3. Wosakhutitsidwa nthawi zambiri
4. Wosakhutitsidwa nkomwe

IUCD

FM15. Kodi alipo mwa abwenzi anu amene anagwiritsapo ntchito lupu (intrauterine contraceptive device)? *(If value = 1(No), Skip to FM18)*

1. Ayi
2. Inde

FM16. Kodi bwenzi lanu linagwiritsapo ntchito lupu pa mwezi wapitawu?

1. Ayi
2. Inde
3. Sindikudziwa
4. Sindinakhalepo ndi bwenzi pa mwezi wathawu

FM17. Kodi munali wokhutitsidwa bwanji inu ndi bwenzi lanu pogwiritsa ntchito lupu?

1. Wokhutitsidwa kwambiri
2. Wokhutitsidwa nthawi zambiri
3. Wosakhutitsidwa nthawi zambiri
4. Wosakhutitsidwa nkomwe

OTHER

FM18. Kodi mukudziwapo za njira ina ili yonse ya kulera imene ingagwiritsidwe ntchito mutagonana opanda chitetezo poteteza mimba?

1. Ayi *(Skip to FM22)*
2. Inde

FM19. Kodi ndinjira yiti yakulera imene munamvapo kuti ingakutetezeni kutenga mimba mutatha kugonana osaziteteza? *(Sankhani zonse zimene zili zoyenera*)

1. Kutawasa
2. Kuika zitsamba m’njira ya abambo
3. Mapilisi a kulera
4. Lupu
5. Kutayira umuna pansi
6. Kondomu
7. Jakisoni
8. Jadelle
9. Zina:________________________________

FM20. Kodi bwezi lanu lidayamba litagwiritsapo ntchito ina mwa njira zapamwambazi mutagonana poteteza mimba?

1. Ayi *(Skip to FF22)*
2. Inde

FM21. Kodi inu kapena bwenzi lanu munayamba mwagwiritsapo imodzi mwa njira zina zakulerazi? *(Sankhani zonse zimene zili zoyenera)*

1. Kutawasa
2. Kuika zitsamba m’njira ya abambo
3. Mapilisi a kulera
4. Lupu
5. Kutayira umuna pansi
6. Kondomu
7. Jakisoni
8. Jadelle
9. Zina:______________________________

FM22. Kodi inu kapena bwenzi lanu munayamba mwagwiritsapo imodzi mwa njira zina zakulerazi? *(Sankhani zonse zimene zili zoyenera. If value=7 (none of the above), Skip to FM24)*

1. Njira yotchinga khomo la chiberekero (Diaphragm)
2. Njira yopha umuna
3. Yochotsa mimba
4. Yotulutsa kunja umuna
5. Kutseka abambo
6. Kutseka amayi
7. Sindinagwiritsepo ina mwa njirazi

FM23. Kodi inu kapena bwenzi lanu munayamba mwagwiritsapo imodzi mwa njira zina zakulerazi mwezi watha? *(Sankhani zonse zimene zili zoyenera)*

1. Njira yotchinga khomo la chiberekero (Diaphragm)
2. Njira yopha umuna
3. Yochotsa mimba
4. Yotulutsa kunja umuna
5. Kutseka abambo
6. Kutseka amayi
7. Sindinagwiritsepo ina mwa njirazi

***READ: “ Mafunso otsatirawa akufunsa za anthu amene inu komanso okondedwa anu munakambilana nawo panthawi yomwe mumaganizira zogwiritsa ntchito njiraya kulera yomwe mukugwiritsira ntchito padakali pano.***

FM24. Kodi munakambirana ndi okondedwa wanu pa njira yoti mugwiritse nchito?

1. Inde
2. Ayi

FM25. Kodi munakambiranapo ndi alangizi azaumoyo a pa chipatala chanu za njira yoti mugwiritse ntchito?

1. Inde
2. Ayi

FM26. Kodi munakambiranako ndi amphunzitsi a ku midzi pa njira yoti mugwiritse ntchito?

1. Inde
2. Ayi

FM27. Kodi munakambiranako ndi achibale anu kapena anzanu pa za njira yoti mugwiritse ntchito?

1. Inde
2. Ayi

FM28. Kodi munatenga uthenga umenewu kuchokera mu magazini, kanema, internet, sukulu, mabuku kapena radio pa za njira yoti mugwiritse ntchito?

1. Magazini
2. Kanema
3. Internet
4. Sukulu
5. Mabuku
6. Radio
7. Zina: ______________________________

**COMMUNICATION (Section G)**

G1. Pa miyezi isanu ndi Umodzi yapitayi, kodi munalankhulapo ndi ena mwa anzanu ndi a m’mbanja mwanu zokhudza ina mwa mitu imeneyi? *(Sankhani zonse zimene zili zoyenera)*

1. Kugwiritsa ntchito kondomu
2. Kulera
3. Kuyezetsa HIV
4. Kuyezetsa kwa matenda ena opatsirana pogonana
5. Palibe

G2. Kodi munalankhulanapo ndi wina mwa abwenzi anu zokhudza ina mwa mitu imeneyi? *(Sankhani zonse zimene zili zoyenera)*

1. Kutenga mimba
2. Kugwiritsa ntchito makondomu
3. Kugwiritsa ntchito kulera
4. Kuyesedwa kwa kachirombo ka HIV
5. Kuyesedwa kwa matenda ena opatsilana pogonana
6. Kungogonana wina ndi mzake
7. Palibe pa zimene zatchulidwazi

G3. Kodi munayamba mwapereka kachirombo ka HIV kwa bwenzi lanu? *(Zimene mukudziwa)*

1. Ayi
2. Inde

G4. Kodi munayamba mwagonana opanda chitetezo musanawauza a bwenzi anu zokhudza m’mene mulili pankhani ya HIV?

1. Ayi
2. Inde

G5. Kodi munayamba mwagonana opanda chitetezo ndi bwenzi lanu ngakhale anadziwa za m’mene mulili pa nkhani ya HIV?

1. Ayi
2. Inde

****READ: “Mafunso otsatirawa ndi okhudza mtokoma (communication).”***

G6. Muli ndi chikhulupiliro chotani kuti mungathe kuuza bwenzi lanu kuti likayesedwe HIV?

1. Ndikukhulupilira kwambiri
2. Ndikukhulupilira
3. Sindikukhulupilira
4. Ndikukaikira kwambiri
5. Sizikugwilizana (bwezi ali ndi kachirombo ka HIV)

G7. Mukanapatsidwa mpata wosankha, mungakonde kuti bwenzi lanu liuzidwe bwanji kuti likayesedwe ndi kupatsidwa chithandizo cha HIV?

1. Kumuuza ndeka
2. Kukhala ndi wopereka chisamaliro (dokotala, namwino, wophunzitsa za umoyo) kuti amuuze
3. Kupereka chikalata chotumizidwa
4. Ndingakonde kuti auzidwe
5. Sizikugwirizana (bwenzi ali ndi kachirombo ka HIV)

G8. Kodi mukuganiza kuti bwenzi lanu lingakuuzeni litakhala ndi matenda opatsirana pogonana?

1. Inde
2. Ayi

G9. Pa ubwenzi wanu, ndi ndani amene nthawi zambiri amapanga/anapanga chiganizo chotenga mimba?

1. Ndine ndimapanga (ndinapanga)
2. Bwenzi langa amapanga ( anapanga)
3. Tonse timapanga mofanana

G10. Pa ubwenzi wanu, ndi ndani amene nthawi zambiri amapanga/anapanga chiganizo chogwiritsa ntchito njira ya kulera?

1. Ndine ndimapanga (ndinapanga)
2. Bwenzi langa amapanga( anapanga)
3. Tonse timapanga mofanana

G11. Pa ubwenzi wanu, ndi ndani amene nthawi zambiri amapanga/anapanga chiganizo chogwiritsa ntchito makondomu?

1. Ndine ndimapanga (ndinapanga)
2. Bwenzi langa amapanga( anapanga)
3. Tonse timapanga mofanana

G12. Pa ubwenzi wanu, ndi ndani amene nthawi zambiri amapanga/anapanga chiganizo choyesa matenda opatsirana pogonana?

1. Ndine ndimapanga (ndinapanga)
2. Bwenzi langa amapanga( anapanga)
3. Tonse timapanga mofanana

G13. Kodi mungathe kupewa kugonana pa nthawi imene simukufuna kutero?

1. Ayi
2. Inde

G14. Kodi mungathe kugwiritsa ntchito kondomu pa nthawi ili yonse imene mukugonana?

1. Ayi
2. Inde

G15. Kodi mungathe kukana kuti musagonane ngati bwenzi lanu silikufuna kugwiritsa ntchito kondomu?

1. Ayi
2. Inde

**ART KNOWLEDGE/USE** **(SECTION H)**

***READ: “Mafunso otsatirawa adzafunsa zokhudza mankhwala anu a HIV. Kutiuza ife za zenizeni zimene munakumana nazo pogwiritsa ntchito mankhwala a HIV (mwachitsanzo kuvuta kwake) zidzatithandiza ife kumvetsetsa bwino zimene zikufunika kuti chisamaliro cha HIV chikhale chabwino.”***

H1. Kodi munayamba mwamwapo mankhwala a HIV (amene amatchedwanso kuti ma ARV kapena ART kapena HAART)? *(If value =1(No), Skip to H5)*

1. Ayi
2. Inde

H2. Kodi pakali pano mukumwa mankhwala a HIV? *(If value = 1(No), Skip to H6)*

1. Ayi
2. Inde

H3. Kodi mwakhala mukumwa mankhwalawa othandiza HIV kwa nthawi yaitali bwanji HIV?

1. Osatha chaka
2. Chaka chimodzi kufikira ziwiri
3. Zaka ziwiri kufikira zisanu
4. Zaka zisanu kufikira khumi
5. Kupitilira zaka khumi

H4. Kodi mukuona kuti thanzi lanu lili bwino kapena likulowa pansi chiyambire kumwa mankhwala a HIV?

1. Lili bwino
2. Silili bwino
3. Palibe kusintha

h5. Kodi kumwa makhwala a kachirombo ka HIV kumathandiza bwanji chiopsyezo chimene chimakhalapo pomupatsira bwenzi lanu ka chirombo ka HIV?

1. Chiopsyezo chimakula
2. Chiopsyezo chimakhala chimodzimodzi
3. Chiopsyezo chimachepa

h6. **FEMALES ONLY:** Kodi kumwa mankhwala a HIV (Panthawi yomwe uli woyembekezera) kumakhudza bwanji chiopsyezo chimene chimakhalapo pomupatsira mwana wobadwayo kachirombo ka HIV

1. Chiopsyezo chimakula
2. Chiopsyezo chimakhala chimodzimodzi
3. Chiopsyezo chimachepa

H7. Kodi simugwiritsa kondomu mukamamwa ma ARV?

1. Ayi
2. Inde
3. Sindikudziwa

H8. Kodi simugwiritsa ntchito njira ya kulera mukamamwa ma ARV?

1. Ayi
2. Inde
3. Sindikudziwa

****READ: “Mwamaliza kafukufuku! Zikomo kwambiri chifukwa chotenga nawo mbali!”***

Q5. Enter survey stop time: ____________

Q6. Enter total survey time: ____________ minutes
