# Supplementary material for: Factors associated with desired fertility among HIV-positive women and men attending two urban clinics in Lilongwe, Malawi
Source: PLoS One. 2018 Jun 13;13(6):e0198798. doi: 10.1371/journal.pone.0198798 (PMC5999219; doi:10.1371/journal.pone.0198798)
Supplement: S2 Text — (DOCX) [file pone.0198798.s002.docx]

**Exploring the Impact of HIV and ART on Knowledge, Attitudes and Practices in Reproductive Health in Lilongwe, Malawi**

**QUANTITATIVE SURVEY**

**DATA ENTRY (SECTION Q)**

Q1. Enter the Participants ID Number: __________________________

Q2. Enter the Date (DD/MM/YYYY): ____________________________

Q3. Enter Research Assistant Name/Code: _______________________

Q4. Enter the survey start time: _________

**DEMOGRAPHICS (SECTION Z)**

Z1. What is your gender?

1. Female
2. Male

Z2. How old are you?

_____ years old *(Write “99” if “Don’t Know”)*

Z3. What is the highest level of education that you have completed?

1. No education
2. Some primary school
3. Completed primary school
4. Some secondary school
5. Completed secondary school
6. More than secondary school

Z4. Which of the following BEST describes your tribal background? Are you. . .

1. Chewa
2. Lambya
3. Lomwe
4. Mang’anja
5. Ndali
6. Ngoni
7. Nkhonde
8. Sena
9. Tonga
10. Tumbuka
11. Yao
12. Other ___________________________________________

Z5. What is your religion?

1. Protestant
2. Catholic
3. Muslim
4. Traditional
5. No religion

Z6. Where do you live? Is your current residence urban or rural?

1. Urban
2. Rural

Z7. How long does it take you on average to get to clinic?

1. Less than 30 minutes
2. 30 minutes to 1 hour
3. 1-2 hours
4. 2-4 hours
5. More than 4 hours

Z8. On average, how many hours per week do you work for income?

1. None, I do not work for income
2. <10 hours per week
3. 10-20 hours per week
4. 20-40 hours per week
5. >40 hours per week

Z9. What material is the floor of your current home made of?

1. Earth/sand/dung
2. Cement
3. Other (specify): ___________________________

**HIV BACKGROUND (sECTION A)**

A1. In what year were you diagnosed with HIV? (Give an estimated year if unsure)

_______ (Write “9999” if “Don’t Know”)

A2. -> Calculate from Age in Question Z2: How old was participant when he/she first learned that he/she was HIV+? (Give an estimated age if unsure; if born with HIV, enter 0)

______ years old *(Write “99” if “Don’t Know”)*

**STIs & CONDOM USE (SECTION B)**

STIs

B1. In the past month, did you worry about getting a sexually transmitted infection?

1. No
2. Yes

B2. Have you ever been diagnosed with a sexually transmitted infection other than HIV?

1. No
2. Yes
3. Do not know

B3. Have you been diagnosed with a sexually transmitted infection (other than HIV) in the last *month*?

1. No
2. Yes
3. Do not know

CONDOM USE

B4. Have you ever used a condom? *(If value=1(No), skip to B8)*

1. No
2. Yes

B5. Did you use condoms in the last month?

1. No
2. Yes

B6. If Yes, then how often?

1. Some of the time

2. All of the time

b6. Did you use a condom the last time you had sex?

1. No
2. Yes

B7. what was the main reason you used a condom the last you had sex? (*Select ONE response only*)

1. To prevent pregnancy
2. To prevent getting an std from my partner(s)
3. To prevent giving/getting HIV to/from my partner(s)

B8. How effective is using a condom at preventing HIV infection?

1. Very effective
2. Somewhat effective
3. Not very effective

B9. In a relationship, which partner can initiate use of a condom?

- - - 1. The man
      2. The woman
      3. Both can initiate condom use

B10. Have you ever been in a relationship where you wanted to use a condom but your partner refused or threatened you? (Such as threatened to break up with you)

1. No
2. Yes

**SEXUAL HISTORY AND CURRENT SEXUAL BEHAVIOR** **(SECTION C)**

C1. Which best describes your current relationship status? (If value 3-5, skip to C3)

1. Married
2. In a committed relationship with only one partner
3. Dating one or more people
4. Single and interested in dating but not currently dating
5. Single and not interested in a relationship at all

C2. How long have you been with your *current* partner?

1. 3 months or less
2. 3-6 months
3. 6 months to 1 year
4. 1 year to 2 years
5. 2 years to 4 years
6. >4 years

C3. Can you describe your relationship with the most recent sexual partner (someone you have oral, vaginal or anal sex with)?

1. Marital partner
2. Other regular partner
3. Casual acquaintance
4. Some you just met
5. Commercial sex worker

6. Other, specify ___________________

C4. Do you think your most recent sexual partner has had sex with other people while you were together?

1. No
2. Yes
3. I do not know

***READ: “The next group of questions is about sexual activity.***

***When a question says ‘any kind of sex’, it is asking about any and all of the following acts: oral sex, vaginal sex and anal sex. Any other time, the question will specify the type of sexual behavior being referred to. By sexual partners we mean someone with whom you had oral, vaginal, or anal sex.***

***For questions that ask for a number, if you are unsure of the exact number, please give an estimated number.”***

C5. How old were you the first time you had any kind of sex? *(For sex, we mean oral, vaginal or anal sex)*

_______ years old

C6. How old were you the first time you had vaginal sex?

_______ years old

C7. How many sexual partners have you ***ever*** had? Count every person you’ve ever been with, even those you had any kind of sex with only once. If you are unsure, estimate the best that you can.

1. 1 partner
2. 2-4 partners
3. 5-10
4. 11-25
5. More than 25 partners

C8. In the past ***month***, how many sexual partners have you had? Count every person, even those you had any kind of sex with only once.

1. 1 partner
2. 2-4 partners
3. 5-10
4. 11-25
5. More than 25 partners
6. No partners in the past month

C9. In the past *month*, how frequently have you had any kind of sex?

1. Have not had sex in the past month
2. One time in the last month
3. Two times in the last month
4. About once a week (3-4 times)
5. Several times a week (more than 4 times)

C10. Of the partners you have had in the past month, how many of your sexual partners do you think knew that you are HIV positive?

1. None
2. Some
3. All

C11. During the past month, how many of your sexual partners did you know were HIV+?

1. None
2. Some
3. All

C12. Was/is your *most recent* sexual partner HIV positive?

1. No
2. Yes
3. Do not know

C13. Was/is your most recent sexual partner aware of your HIV status?

1. No
2. Yes
3. Do not know

C14. At any time in your life, has someone ever forced you to have oral, vaginal, or anal sex against your will?

1. No
2. Yes

C15. At any time in your life, has someone physically abused you (beat, punched, slapped)?

1. No
2. Yes

C16. Have you ever had sex in exchange for money, food, drugs, or a place to stay?

1. No
2. Yes

*READ: “The next questions are about drinking alcohol and using drugs. Remember, all your answers are confidential.”*

C17. Have you ever drank alcohol? (If value=1(No), then skip to C19)

1. No
2. Yes

C18. In the past month, how often did you drink alcohol before or during sex?

1. Never
2. Sometimes
3. Always
4. I have not had sex in the past month

C19. Have you ever used drugs (for example marijuana/chamba, khat)? (If value=1(No), then skip to DF1 if female, DM1 if male)

1. No
2. Yes

C20. In the past month, how often did you use drugs before or during sex? (If value=1(No), then skip to DF1 if female, DM1 if male)

1. Never
2. Sometimes
3. Always
4. I have not had sex in the past month

C21. In the past month, what drug(s) did you use prior to having sex? *(Select ALL that apply)*

1. Marijuana/chamba
2. Other (specify): ________
3. I have not used drugs prior to having sex in the past month

**FERTILITY PREFERENCES & PREGNANCY HISTORY (SECTION DF) (FEMALES ONLY)**

***READ: We are now going to ask about pregnancy and* *pregnancy related topics.”***

DF1. Have you ever been pregnant? (If value=1(No), skip to DF15)

1. No
2. Yes

DF2. How many children do you have?

1. No children
2. One
3. Two
4. Three
5. Four
6. Five or more children

DF3. How old were you the FIRST time you got pregnant?

____ years old

DF4. Have you ever had a miscarriage or delivered a stillborn?

1. No
2. Yes

DF5. Were you diagnosed with HIV *before* your most recent pregnancy?

1. No
2. Yes

DF6. Were you diagnosed with HIV *during* your most recent pregnancy?

1. No
2. Yes

DF7. Have you ever had a child born with HIV?

1. No
2. Yes

DF8. Have any of your children died due to HIV/AIDs?

1. No
2. Yes

DF9. Have you ever had an unplanned pregnancy (a pregnancy that was mistimed, unplanned, or unwanted at when you got pregnant)?

1. No
2. Yes

DF10. How many unplanned pregnancies have you had?

________ unplanned pregnancies

DF11. Have you ever had an abortion in the past (for any reason)? (if value=1(No), skip to DF15)

1. No
2. Yes

DF12. Have you ever had an abortion because of your HIV status?

1. No
2. Yes

DF13. What was your partner’s HIV status for the pregnancy (or pregnancies) you aborted?

1. HIV-negative
2. HIV-positive
3. I have ended more than one pregnancy and partners have been both HIV positive and negative
4. I don’t know

DF14. Did your partner’s HIV status influence your ending the pregnancy?

1. No
2. Yes

DF15. In the past 6 months, did you worry about getting pregnant?

1. No
2. Yes

DF16. Do you want or plan to have more children (at any time in the future)? (if value-1(No), skip to DF18)

1. No
2. Yes

DF17. Do you want to get pregnant in the next 2 years?

1. No
2. Yes

DF18. Do you believe that there is pressure for women to have children, even if a woman is HIV positive?

1. No
2. Yes

DF19. If you had an unplanned pregnancy, would you ever consider an abortion?

1. No
2. Yes
3. Do not know

DF20. Do you believe abortion is safe?

1. Yes
2. No
3. Do not know

DF21. Did getting diagnosed with HIV change whether you wanted or planned to have children?

1. No
2. Yes

DF22. If you were HIV-negative, would you want to have more children?

1. No
2. Yes

DF23. In the past 6 months, did you think you may not be able to get pregnant because you are infertile?

1. No
2. Yes

DF24. Does/did your most recent sexual partner want (more) children?

1. No
2. Yes
3. I do not currently have a partner

DF25. Do you feel pressure to have more children from your partner?

1. No
2. Yes

DF26. Do you feel pressure to have more children from your family?

1. No
2. Yes

DF27. Do you feel pressure to have more children from your community?

1. No
2. Yes

DF28. Do you feel that getting pregnant would be healthy or unhealthy for you?

1. Healthy

2. Unhealthy

DF29. In your opinion, do most people disapprove of HIV positive women having children?

1. No
2. Yes

DF30. In your opinion, is it everyone’s right to have a child, regardless of HIV status?

1. No
2. Yes

DF31. Can HIV positive women give birth to HIV negative babies?

1. No
2. Yes

DF32. Do you know if treatments exist that help prevent mother to child transmission of HIV?

1. No
2. Yes

***READ: “Now we would like to know about your discussions about contraception and sex with your doctor and/or other health care workers. Remember, your answers will not be shared with any of your providers. We want to know how you truly feel about your discussions about family planning with your HIV care providers.”***

DF33. Have you felt that health care workers try to discourage you from having children after learning of your HIV diagnosis?

1. No
2. Yes

DF34. Do you feel that you would have the support of your doctor if you decided to have more children?

1. No
2. Yes

DF35. Do you feel that your doctor and/or health care workers have given you enough information to make informed decisions about what contraceptives to use?

1. No
2. Yes

DF36. Have you ever had a discussion as a couple regarding fertility intentions or family planning?

- - - 1. No
      2. Yes

DF37. Do you think it is helpful if couples meet together with a provider or counselor to discuss family planning?

1. No
2. Yes

DF38. Would you be comfortable hearing and talking about family planning in a group setting?

1. No
2. Yes

**FERTILITY PREFERENCES & PREGNANCY HISTORY (SECTION DM) (MALES ONLY)**

*READ: “We are now going to ask about pregnancy and* *pregnancy related topics.”*

DM1. Have you ever fathered a pregnancy? (If value=1(No), skip to DM15)

1. No
2. Yes

DM2. How many children do you have?

1. No children
2. One
3. Two
4. Three
5. Four
6. Five or more children

DM3. How old were you the FIRST time your partner got pregnant?

____ years old

DM4. The last time your partner became pregnant, did you know you were HIV-positive?

1. No
2. Yes

DM5. Have you ever fathered a child that was born with HIV?

1. No
2. Yes

DM6. Have any of your children died due to HIV/AIDs?

1. No
2. Yes

DM7. Have your partner ever had an unplanned pregnancy (a pregnancy that was mistimed, unplanned, or unwanted at when you got pregnant)?

1. No
2. Yes

DM8. In the past 6 months, did you worry about your partner getting pregnant?

1. No
2. Yes

DM9. Do you want or plan to have more children (at any time in the future)? (if value-1(No), skip to DM11)

1. No
2. Yes

DM10. Do you want your partner to get pregnant in the next 2 years?

1. No
2. Yes

DM11. Do you believe that there is pressure for women to have children, even if a woman is HIV positive?

1. No
2. Yes

DM12. If your partner had an unplanned pregnancy, would you ever consider an abortion?

1. No
2. Yes
3. Do not know

DM13. Do you believe abortion is safe?

1. Yes
2. No
3. Do not know

DM14. Did getting diagnosed with HIV change whether you wanted or planned to have children?

1. No
2. Yes

DM15 If you were HIV-negative, would you want to have more children?

1. No
2. Yes

DM16. Does/did your most recent sexual partner want (more) children?

1. No
2. Yes
3. I do not currently have a partner

DM17. Do you feel pressure to have more children from your partner?

1. No
2. Yes

DM18. Do you feel pressure to have more children from your family?

1. No
2. Yes

DM19. Do you feel pressure to have more children from your community?

1. No
2. Yes

DM20. In your opinion, do most people disapprove of HIV positive women having children?

1. No
2. Yes

DM21. In your opinion, is it everyone’s right to have a child, regardless of HIV status?

1. No
2. Yes

DM22. Can HIV positive women give birth to HIV negative babies?

1. No
2. Yes

DM23. Do you know if treatments exist that help prevent mother to child transmission of HIV?

1. No
2. Yes

DM24. Do you feel that your doctor and/or health care workers have given you enough information to make informed decisions about what contraceptives to use?

1. No
2. Yes

DM25. Have you ever had a discussion as a couple regarding fertility intentions or family planning?

1. No
2. Yes

DM26. Do you think it is helpful if couples meet together with a provider or counselor to discuss family planning?

1. No
2. Yes

DM27. Would you be comfortable hearing and talking about family planning in a group setting?

1. No
2. Yes

**CONTRACEPTIVE KNOWLEDGE/ATTITUDES (sECTION EF) (FEMALES ONLY)**

***READ: “With this next set of questions we are going to assess the general level of knowledge and misunderstandings about contraceptives.”***

EF1. Have you heard of any birth control methods having a negative impact on your disease (worsening your HIV)? (If value = 1(No), skip to EF3)

1. No
2. Yes

EF2. Which birth control method(s) have you heard of having a negative impact on your disease (worsening your HIV)? *Pick all that apply.*

1. Condoms
2. Depo Provera
3. Pills
4. IUCD
5. Implant
6. None of the above

Ef3. Which birth control method(s) have you heard that you should not use when taking HIV medications? *Pick ALL that apply.*

1. Condoms
2. Depo Provera
3. Pills
4. IUCD
5. Implant
6. None of the above

EF4. Have you heard that birth control methods make it harder to get pregnant in the future? (If value=1(No), skip to EF6)

1. No
2. Yes

EF5. Which birth control method(s) have you heard make it harder to get pregnant in the future? *(Select ALL that apply)*

1. Condoms
2. Depo Provera
3. Pills
4. IUCD
5. Implant
6. None of the above

EF6. Before today, had you heard about the IUCD? (If value=1(No), skip to EF8)

1. No
2. Yes

EF7. Before today, had you heard about the contraceptive implant, also known as Jadelle, SinoImplant or Norplant?

1. No
2. Yes

EF8. Does tying a rope around a woman’s waist (for example by a traditional birth attendant) prevent pregnancy?

1. No
2. Yes

EF9. Do you think condoms are effective at preventing pregnancy?

1. No
2. Yes

EF10. If you are using a birth control method, do you think you also need to use a condom?

1. No
2. Yes

EF11. Do oils in condoms cause swelling of a woman’s belly?

1. No
2. Yes

Don’t know

EF12. Do injections or pills affect a woman’s sexual desire?

1. Neither injections nor pills affect a woman’s sexual desire
2. Only pills affect a woman’s sexual desire
3. Only injections affect a woman’s sexual desire
4. Both injections and pill affect a woman’s sexual desire
5. Don’t know

EF13. Does a woman taking pills or using injections affect a man’s desire to have sex with her?

1. Neither injections nor pills affect a man’s sexual desire
2. Only pills affect a man’s sexual desire
3. Only injections affect a man’s sexual desire
4. Both injections and pill affect a man’s sexual desire
5. Don’t know

EF14. Do you think that birth control methods that contain hormones cause cancer?

1. No
2. Yes
3. Don’t know

EF15. Which birth control method(s) prevent transmission of HIV to your partner? *(Select ALL that apply)*

1. Depo Provera
2. Pills
3. IUCD
4. Implant
5. Condoms
6. Withdrawal
7. Emergency contraception
8. Abstinence
9. Monogamy
10. None of the above

EF16. Which birth control method(s) *increase* the risk of transmission of HIV to your partner? *(Select ALL that apply)*

1. Depo Provera
2. Pills
3. IUCD
4. Implant
5. Condoms
6. Withdrawal
7. Emergency contraception
8. Abstinence
9. Monogamy
10. None of the above

EF17. Where do you learn about family planning? (*Select ALL that apply).*

1. From family members
2. From friends and/or neighbors
3. From community health workers
4. From nurse at clinic
5. Clinician/doctor at clinic
6. From the Family Planning Association of Malawi (FPAM)
7. From Traditional Birth Attendants
8. From the radio or TV
9. From the newspaper or magazines
10. From other sources, please specify __________

EF18. Where would you prefer to receive information about family planning?

1. At home
2. At a clinic
3. At a community group
4. Radio
5. Other, please specify ______________

EF19. Where would you prefer to obtain your family planning method?

1. At home
2. At a clinic
3. At a health post
4. At a pharmacy
5. Other, please specify _______________

**CONTRACEPTIVE KNOWLEDGE/ATTITUDES (sECTION EM) (MALES ONLY)**

***READ: “With this next set of questions we are going to assess the general level of knowledge and misunderstandings about contraceptives.”***

EM1. Have you heard that birth control methods make it harder to get pregnant in the future? (If value=1(No), skip to EM3)

1. No
2. Yes

EM2. Which birth control method(s) have you heard make it harder to get pregnant in the future? *(Select ALL that apply)*

1. Condoms
2. Depo Provera
3. Pills
4. IUCD
5. Implant
6. None of the above

EM3. Does tying a rope around a woman’s waist (for example by a traditional birth attendant) prevent pregnancy?

1. No
2. Yes

EM4. Do you think condoms are effective at preventing pregnancy?

1. No
2. Yes

EM5. If your partner is using a birth control method, do you think you also need to use a condom?

1. No
2. Yes

EM6. Do injections and pills affect a woman’s sexual desire?

1. Neither injections nor pills affect a woman’s sexual desire
2. Only pills affect a woman’s sexual desire
3. Only injections affect a woman’s sexual desire
4. Both injections and pill affect a woman’s sexual desire
5. Don’t Know

EM7. Does a woman taking pills or using injections affect a man’s desire to have sex with her?

1. Neither injections nor pills affect a man’s sexual desire
2. Only pills affect a man’s sexual desire
3. Only injections affect a man’s sexual desire
4. Both injections and pill affect a man’s sexual desire
5. Don’t know

EM8. Do you believe that birth control methods that contain hormones cause cancer?

1. No
2. Yes
3. Don’t know

EM9. Which birth control method(s) prevent transmission of HIV to your partner? *(Select ALL that apply)*

1. Depo Provera
2. Pills
3. IUCD
4. Implant
5. Condoms
6. Withdrawal
7. Emergency contraception
8. Abstinence
9. Monogamy
10. None of the above

EM10. Which birth control method(s) *increase* the risk of transmission of HIV to a partner? *(Select ALL that apply)*

1. Depo Provera
2. Pills
3. IUCD
4. Implant
5. Condoms
6. Withdrawal
7. Emergency contraception
8. Abstinence
9. Monogamy
10. None of the above

**FEMALE CONTRACEPTIVE USE (SECTION FF)** **(FEMALES ONLY)**

*This section is for women only; men should respond to contraceptive use questions in Section FM instead.*

***READ “We are going to ask about what types of birth control methods (also known as contraceptives), if any, you have used and your experiences with them.”***

FF1. Have you ever used a birth control method? (By birth control we mean using something to not get pregnant such as contraceptive pills, IUCDs, female or male sterilization, etc. (If value = 1(No), skip to G1)

1. No
2. Yes

FF2. Are *currently* using any birth control method?

1. No
2. Yes

FF3. were you using any method of birth control (other than condoms) the last time you had sex? (If value=1(No), then skip to FF5)

1. No
2. Yes

FF4. what methods of birth control did you use the last time you had sex? (*Select ALL that apply*)

1. Condom
2. IUCD
3. Implant
4. Injection (Depo Provera)
5. Pill
6. withdrawal
7. Emergency Contraception
8. Female sterilization/ tubal ligation
9. Male sterilization/ vasectomy
10. Other, please specify: _____________________________________

FF5. Does your religion play a role in deciding whether or not you use birth control?

1. No
2. Yes

FF6. Do any medications you take play a role in deciding whether or not you use birth control?

1. No
2. Yes
3. Not applicable (I am not on any medications)

ORAL CONTRACEPTIVES

FF7. Have you ever used the birth control pill? (If value = 1(No), skip to FF12 [Depo provera])

1. No
2. Yes

FF8. what is the longest time you used the pill?

1. Less than 1 month
2. 1-3 months
3. 4-6 months
4. 7-12 months
5. More than 1 year

FF9. Did you use the pill in the last month?

1. No
2. Yes

FF10. How satisfied are/were you with using the pill?

1. Very satisfied
2. Mostly satisfied
3. Mostly dissatisfied
4. Very dissatisfied

FF11. when you used the pill, did you experience any of the following? *(Select ALL that apply)*

1. Periods got worse
2. Periods got better
3. Periods went away
4. Headaches
5. Weight gain
6. Hair loss
7. Improved skin (Less acne)
8. Didn’t have any of these

DEPO PROVERA

FF12. Have you ever used Depo Provera (shot)? (If value = 1(No), skip to F18 [Implant])

1. No
2. Yes

FF13. what is the longest period of time you used Depo?

1. Less than 1 month
2. 1-3 months
3. 4-6 months
4. 7-12 months
5. More than 1 year

FF14. Did you get your last Depo shot when you were scheduled to?

1. Yes
2. No
3. It was my first shot

FF15. Did you use Depo in the past month?

1. No
2. Yes

FF16. How satisfied are/were you with using Depo?

1. Very satisfied
2. Mostly satisfied
3. Mostly dissatisfied
4. Very dissatisfied

FF17. when you used Depo, did you experience any of the following? *(Select ALL that apply)*

1. Periods got worse
2. Periods got better
3. Periods went away
4. Headaches
5. weight gain
6. Hair loss
7. Improved skin (Less acne)
8. Didn’t have any of these

IMPLANT

FF18. Have you ever used a contraceptive implant (norplant, jadelle or sinoimplant)? (If value = 1(No), skip to FF23 [IUCD])

1. No
2. Yes

FF19. what is the longest period of time you used the implant?

1. Less than 1 month
2. 1-3 months
3. 4-6 months
4. 7-12 months
5. More than 1 year

FF20. Did you use the implant in the past month?

1. No
2. Yes

FF21. How satisfied are/were you with using the implant?

1. Very satisfied
2. Mostly satisfied
3. Mostly dissatisfied
4. Very dissatisfied

FF22. when you had the implant, did you experience any of the following? *(Select ALL that apply)*

1. Periods got worse
2. Periods got better
3. Periods went away
4. Headaches
5. Weight gain
6. Hair loss
7. Weight loss
8. Improved skin (Less acne)

IUCD

FF23. Have you ever used the IUCD? (If value = 1(No), skip to FF28 [Other])

1. No
2. Yes

FF24. what is the longest period of time you used the IUCD?

1. Less than 1 month
2. 1-3 months
3. 4-6 months
4. 7-12 months
5. More than 1 year

FF25. Did you use the IUCD the past month?

- - - 1. Yes
      2. No

FF26. How satisfied are/were you with using the IUCD?

1. Very satisfied
2. Mostly satisfied
3. Mostly dissatisfied
4. Very dissatisfied

FF27. when you had an IUCD, did you experience any of the following? *(Select ALL that apply)*

1. Periods got worse
2. Periods got better
3. Periods went away
4. Headaches
5. Weight gain
6. Hair loss
7. Weight loss
8. Improved skin (Less acne)
9. Didn’t have any of these

OTHER

FF28. Are you aware of any form of birth control that can be used after unprotected intercourse to prevent a pregnancy? *(If value=1, skip to FF32)*

1. No
2. Yes

FF29. Which of the following methods have you heard will prevent pregnancy if used after intercourse? *(Select ALL that apply)*

- - - 1. Douching
      2. Putting herbs in vagina
      3. Birth control pills
      4. IUCD
      5. Withdrawal
      6. Condoms
      7. Injections
      8. Implant
      9. Other __________________________

FF30. Have you ever used any of these methods after intercourse to prevent pregnancy?

1. No *(Skip to FF32)*
2. Yes

FF31. Which of the following methods have you used after intercourse to prevent pregnancy? *(Select ALL that apply)*

1. Douching
2. Putting herbs in vagina
3. Birth control pills
4. IUCD
5. Withdrawal
6. Condoms
7. Injection
8. Implant
9. Other __________________________

FF32. Have you ever used any of these other methods of birth control? *(Select ALL that apply*. *If value=7 (none of the above), skip to FF34)*

1. Diaphragm
2. spermicides
3. Abortion
4. Withdrawal
5. Vasectomy
6. Tubal ligation
7. None of the above

FF33. Have you used any of these other methods in the last month? *(Select ALL that apply)*

1. Diaphragm
2. spermicides
3. Abortion
4. Withdrawal
5. Vasectomy
6. Tubal ligation
7. I haven’t used any of these

***READ: “The next set of questions is about to the sources you used when deciding to use your most recent birth control method.”***

FF34. When deciding to use your most recent birth control method, did you talk to your partner about which method to use?

1. Yes
2. No

FF35. When deciding to use your most recent birth control method, did you talk to any health care providers at your health centre about which method to use?

1. Yes
2. No

FF36. When deciding to use your most recent birth control method, did you talk to any community-based educators about which method to use?

1. Yes
2. No

FF37. When deciding to use your most recent birth control method, did you talk to any other family or friends about which method to use?

1. Yes
2. No

FF38. When deciding to use your most recent birth control method, did you get information from magazines, TV, the internet, school, books, radio about which method to use?

1. Magazines
2. TV
3. Internet
4. School
5. Books
6. Radio
7. Other (specify):_________ __________________

****Skip to G1 after completing this section.***

**MALE CONTRACEPTIVE USE (SECTION FM) (FOR MALES ONLY)**

***READ: “Now we are going to ask about what types of birth control methods (also known as contraceptives), if any, your partner(s) has used and your experiences with them.”***

FM1. Have you or any of your partners ever used birth control? (By birth control we mean using something to not get pregnant such as condoms, pills, injections, or sterilization/vasectomy (If value = 1(No), skip to G1])

1. No
2. Yes
3. Do not know

FM2. Are you or your partner *currently* using any birth control method?

1. No
2. Yes

FM3. Were you or your partner using any method of birth control the last time you had sex? (If value=1(No), then skip to FM5)

1. No
2. Yes

FM4. what methods of birth control did you and your partner use the last time you had sex? *(Select ALL that apply)*

1. Condoms
2. IUCD
3. Implant
4. Injection
5. Pill
6. withdrawal
7. Emergency Contraception
8. Male sterilization/vasectomy
9. Female Sterilization/tubal ligation
10. Other, please specify: _________________________

FM5. Does your religion play a role in deciding whether or not you and your partner use birth control?

1. No
2. Yes

OCPs

FM6. Have any of your partners you ever used the pill? (If value = 1(No), skip to FM9 [Depo provera])

1. No
2. Yes
3. Do not know

FM7. Have any of your partners used the pill in the last month?

- - - 1. No
      2. Yes
      3. Do not know
      4. Have not had a partner in past month

FM8. How satisfied are/were you with your partner(s) using the pill?

1. Very satisfied
2. Mostly satisfied
3. Mostly dissatisfied
4. Very dissatisfied

DEPO PROVERA

FM9. Have any of your partners ever used Depo Provera (shot)? (If value = 1(No), skip to FM12 [Implant])

1. No
2. Yes
3. Do not know

FM10. Did your partner use Depo in the past month?

1. No
2. Yes
3. Do not know
4. Have not had a partner in past month

FM11. How satisfied are/were you with your partner using Depo?

1. Very satisfied
2. Mostly satisfied
3. Mostly dissatisfied
4. Very dissatisfied

IMPLANT

FM12. Have any of your partners ever used a contraceptive implant (norplant, jadelle or sinoimplant)? (If value = 1(No), skip to FM15 [IUCD])

1. No
2. Yes
3. Do not know

FM13. Did your partner use the implant in the past month?

1. No
2. Yes
3. Do not know
4. Have not had a partner in past month

FM14. How satisfied are/were you with your partner using the implant?

1. Very satisfied
2. Mostly satisfied
3. Mostly dissatisfied
4. Very dissatisfied

IUCD

FM15. Have you had a any partners who used the IUCD (intrauterine contraceptive device)? (If value = 1(No), skip to FM18)

1. No
2. Yes

FM16. Has your current partner used the IUCD the past month?

1. No
2. Yes
3. Do not know
4. Have not had a partner in past month

FM17. How satisfied are/were you with your partner using the IUCD?

1. Very satisfied
2. Mostly satisfied
3. Mostly dissatisfied
4. Very dissatisfied

OTHER

FM18. Are you aware of any form of birth control that can be used after unprotected intercourse to prevent a pregnancy?

1. No *(Skip to FM22)*
2. Yes

FM19. Which of the following methods have you heard will prevent pregnancy if used after intercourse? *(Select ALL that apply)*

- - - 1. Douching
      2. Putting herbs in vagina
      3. Birth control pills
      4. IUCD
      5. Withdrawal
      6. Condoms
      7. Injection
      8. Implant
      9. Other __________________________

FM20. Have any of your partners ever used any of these methods after intercourse to prevent pregnancy?

- - - 1. No *(Skip to FF22)*
      2. Yes

FM21. Which of the following methods have your partners used after intercourse to prevent pregnancy? *(Select ALL that apply)*

1. Douching
2. Putting herbs in vagina
3. Birth control pills
4. IUCD
5. Withdrawal
6. Condoms
7. Injection
8. Implant
9. Other __________________________

FM22. Have you or your partner ever used any of these other methods of birth control? *(Select ALL that apply*. (If value=7 (none of the above), skip to FM24)

1. Diaphragm
2. spermicides
3. Abortion
4. Withdrawal
5. Vasectomy
6. Tubal Ligation
7. None of the above

FM23. Have you or your partner ever used any of these other methods in the last month? *(Select ALL that apply)*

1. Diaphragm
2. spermicides
3. Abortion
4. Withdrawal
5. Vasectomy
6. Tubal Ligation
7. None of the above

***READ: “The next set of questions is about who you talked to when you and your partner were deciding to use your most recent birth control method.”***

FM24. Did you talk to your partner about which method to use?

1. Yes
2. No

FM25. Did you talk to any health care providers at your health centre about which method to use?

1. Yes
2. No

FM26. Did you talk to any community-based educators about which method to use?

1. Yes
2. No

FM27. Did you talk to any other family or friends about which method to use?

1. Yes
2. No

FM28. Did you get information from magazines, TV, the internet, school, radiobooks about which method to use?

1. Magazines
2. TV
3. Internet
4. School
5. Books
6. Radio
7. Other (specify):_________ __________________

**COMMUNICATION (Section G)**

G1. In the past 6 months, did you talk with any *friends and* *family members* about any of these subjects? (*Check ALL that apply).*

1. Condom use
2. Birth control
3. HIV testing
4. Other STD testing
5. None of the above

G2. Have you ever talked with any of your *partners* about any of these subjects? *Check ALL that apply.*

1. Pregnancy
2. whether to use condoms
3. whether to use birth control
4. whether to get tested for HIV
5. Whether to get tested for other STDs
6. whether to only have sex with each other
7. None of the above

G3. Have you ever transmitted HIV to a sexual partner? (That you know of)

1. No
2. Yes

G4. Have you ever had unprotected sex without telling your partner about your HIV status?

1. No
2. Yes

G5. Have you ever had unprotected sex with your partner even though he/she was aware of your HIV status?

1. No
2. Yes

****READ: The next series of questions is about communication with your most recent steady partner.***

G6. How sure are you that you could ask your partner to get tested for HIV?

1. Very sure
2. sure
3. Not sure
4. Very unsure
5. Not applicable (partner is known to be HIV positive)

G7. If you were given the choice, how would you want your partner to be told that he/she should get tested and treated for HIV?

1. Tell him/her myself
2. Have a provider (doctor, nurse, health educator) tell him/her
3. Give a referral sheet
4. I wouldn’t want him/her to be told
5. Not applicable (partner is known to be HIV positive)

G8. Do you think your most recent sexual partner would tell you if he/she had an STD?

1. Yes
2. No

G9. In your relationship, who usually makes (or made) decisions about whether to get pregnant?

1. I do (did)
2. My partner does (did)
3. we both do (did) equally

G10. In your relationship, who usually makes (or made) decisions about whether to use birth control?

1. I do (did)
2. My partner does (did)
3. we both do (did) equally

G11. In your relationship, who usually makes (or made) decisions about using condoms?

1. I do (did)
2. My partner does (did)
3. we both do (did) equally

G12. In your relationship, who usually makes (or made) decisions about getting tested for STDs?

1. I do (did)
2. My partner does (did)
3. we both do (did) equally

G13. Would you be able to avoid sex any time you did not want it?

1. No
2. Yes

G14. Would you be able to use a condom every time you have sexual intercourse?

1. No
2. Yes

G15. Would you be able to refuse to have sex if your partner did not want to use a condom?

1. No
2. Yes

**ART KNOWLEDGE/USE** **(SECTION H)**

***READ: “The next questions will ask about your HIV medications. Telling us about your actual experiences with HIV medications (for example how hard it is) will help us to get a better understanding of what is needed to improve HIV care.”***

H1. Have you ever taken HIV medications (also known as antiretroviral medications or ART or HAART)? (If value =1(No), skip to H5)

1. No
2. Yes

H2. Are you *currently* taking HIV medications? (If value = 1(No), skip to H6)

1. No
2. Yes

H3. How long have you been taking medication for treating HIV?

1. Less than one year
2. 1 to 2 years
3. 2 to 5 years
4. 5 to 10 years
5. Over 10 years

H4. Has your overall health status gotten better or worse since beginning HIV medications?

1. Gotten better
2. Gotten worse
3. No change

h5. How does taking HIV medications affect the risk of giving your strain of HIV to your partner?

1. Increases risk
2. Risk stays the same
3. Decreases risk

H6. **FEMALES ONLY:** How does taking HIV medications (during pregnancy) affect the risk of a mother passing on HIV to a new baby?

1. Increases risk
2. Risk stays the same
3. Decreases risk

H7. Are you less likely to use a condom if you are taking your ART drugs?

1. No
2. Yes
3. Do not know

H8. Are you less likely to use a family planning method if you are taking your ART drugs?

1. No
2. Yes
3. Do not know

****READ: “You’re all done with the survey! Thank you very much for your participation!”***

Q5. Enter survey stop time: ____________

Q6. Enter total survey time: ____________ minutes
