# Supplementary material for: Factors associated with desired fertility among HIV-positive women and men attending two urban clinics in Lilongwe, Malawi
Source: PLoS One. 2018 Jun 13;13(6):e0198798. doi: 10.1371/journal.pone.0198798 (PMC5999219; doi:10.1371/journal.pone.0198798)
Supplement: S1 Fig — (PPTX) [file pone.0198798.s003.pptx]

## Slide 1
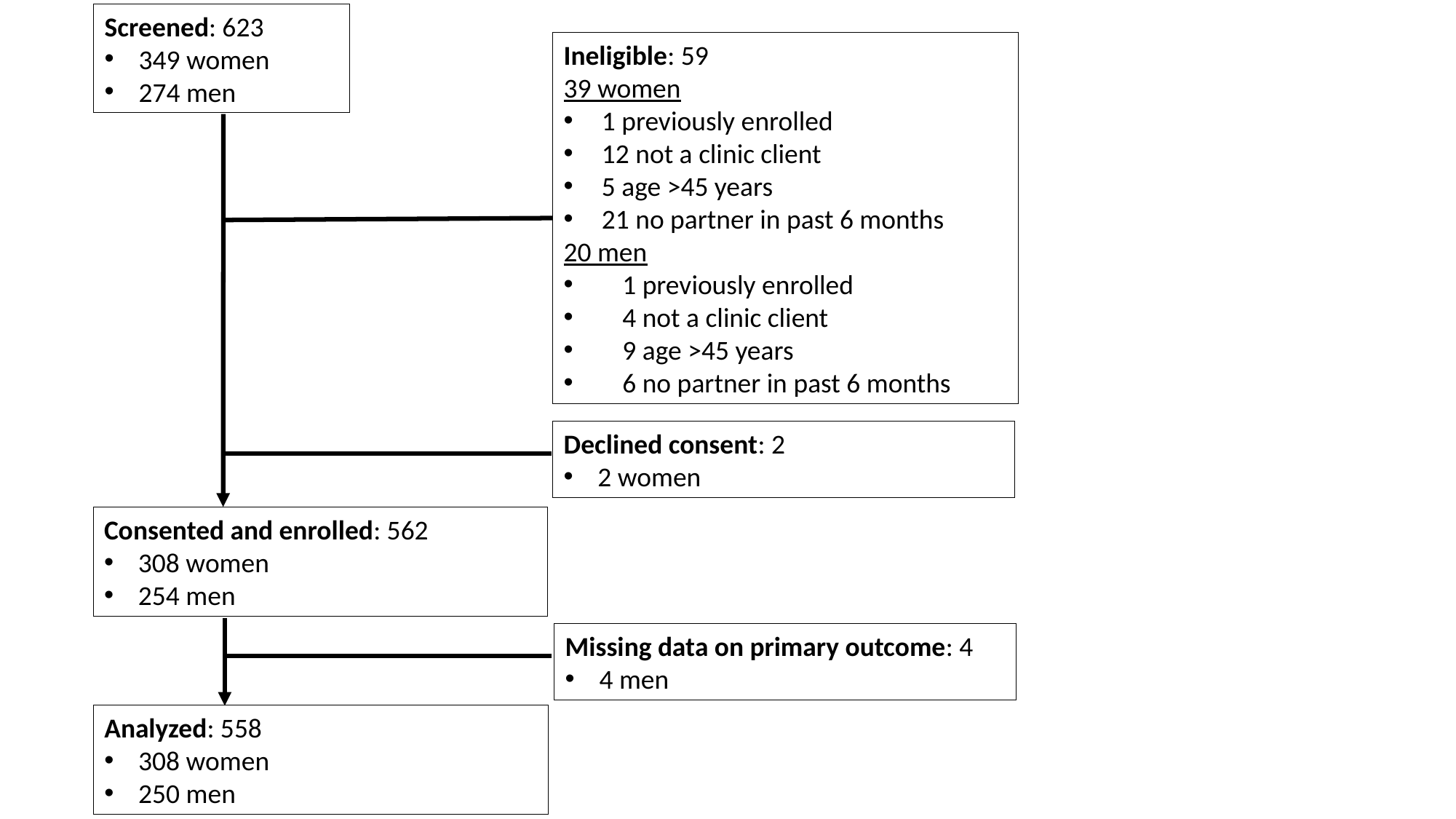

Screened: 623
349 women
274 men
Ineligible: 59
39 women
 1 previously enrolled
 12 not a clinic client
 5 age >45 years
 21 no partner in past 6 months
20 men
 1 previously enrolled
 4 not a clinic client
 9 age >45 years
 6 no partner in past 6 months
Declined consent: 2
2 women
Consented and enrolled: 562
308 women
254 men
Missing data on primary outcome: 4
4 men
Analyzed: 558
308 women
250 men
