# Supplementary material for: Factors associated with desired fertility among HIV-positive women and men attending two urban clinics in Lilongwe, Malawi
Source: PLoS One. 2018 Jun 13;13(6):e0198798. doi: 10.1371/journal.pone.0198798 (PMC5999219; doi:10.1371/journal.pone.0198798)
Supplement: S1 Table — (DOC) [file pone.0198798.s004.doc]

S1 Table. Bivariable characteristics associated with desire for fertility among women and men who receive care at large, public HIV clinics in Lilongwe, Malawi

|  |  | Women | | | Men | | |
| --- | --- | --- | --- | --- | --- | --- | --- |
|  |  | Desire fertility | | | Desire fertility | | |
| Characteristic |  | Yes  (n=105)  n(%) or  mean (SD) | No  (n=203)  n(%) or  mean (SD) | p | Yes  (n=70)  n(%) or  mean (SD) | No  (n=180)  n(%) or  mean (SD) | p |
| Would desire fertility if were counterfactually HIV negative |  | 86 (46) | 101 (54) | <0.01 | 61 (45) | 76 (55) | <0.01 |
| Sociodemographic | | | | | | | |
| Age |  | 29.7 (6.4) | 33.7 (6.2) | <0.01 | 35.3 (5.5) | 37.7 (5.2) | <0.01 |
| Urban residence (*missing 1 woman)* |  | 96 (35) | 180 (65) | 0.52 | 55 (26) | 158 (74) | 0.07 |
| Education: completed secondary school |  | 18 (35) | 33 (65) | 0.84 | 12 (21) | 44 (79) | 0.21 |
| Religion (*missing 1 woman & 1 man)* | Catholic | 27 (44) | 35 (56) | 0.08 | 13 (25) | 38 (75) | 0.69 |
|  | Not catholic | 78 (32) | 167 (68) |  | 56 (28) | 142 (72) |  |
| HIV-related |  |  |  |  |  |  |  |
| Years since HIV diagnosis (*missing 3 women)* |  | 4.1 (3.4) | 4.9 (3.4) | 0.04 | 5.0 (4.3) | 4.2 (4.0) | 0.15 |
| Currently taking ART |  | 89 (33) | 184 (67) | 0.12 | 61 (28) | 158 (72) | 0.89 |
| Health status since beginning ART^a^ | Improved | 83 (33) | 169 (67) | 0.75 | 58 (28) | 147 (72) | 1.0 |
|  | Worsened | 6 (33) | 12 (67) |  | 3 (25) | 9 (75) |  |
|  | No change | 0 (0) | 3 (100) |  | 0 (0) | 2 (100) |  |
| Sexual characteristics and risk behaviors | | | | | | | |
| Married or in monogamous relationship |  | 104 (35) | 196 (65) | 0.27 | 68 (28) | 178 (72) | 0.31 |
| Most recent partner’s HIV status (*missing 1 woman & 1 man)* | Negative | 22 (38) | 36 (62) | 0.36 | 8 (18) 51 (28) | 36 (82) | 0.07 |
|  | Positive | 82 (35) | 167 (65) |  | 51 (28) | 132 (72) |  |
|  | Unknown | 14 (26) | 40 (74) |  | 10 (45) | 12 (55) |  |
| Length of time with current partner *(missing 8 women & 3 men)* | < 4 years | 48 (48) | 51 (52) | <0.01 | 34 (45) | 41 (55) | <0.01 |
|  | > 4 years | 56 (28) | 145 (72) |  | 34 (20) | 138 (80) |  |
| Who decides whether to use contraception *(missing 25 women & 27 women)* | Woman does | 62 (31) | 141 (69) | 0.60 | 25 (28) | 64 (72) | 0.33 |
|  | Man does or both do | 27 (34) | 53 (66) |  | 30 (22) | 104 (78) |  |
| Reproductive history | | | | | | | |
| Number of children | 0 | 17 (74) | 6 (26) | <0.01 | 4 (80) | 1 (20) | <0.01 |
|  | 1 | 38 (58) | 27 (42) |  | 26 (65) | 14 (35) |  |
|  | 2-3 | 39 (27) | 107 (73) |  | 26 (33) | 87 (77) |  |
|  | 4 or more | 11 (15) | 63 (85) |  | 14 (15) | 78 (85) |  |
| Has a child born with HIV (*missing 1 woman)* |  | 13 (22) | 47 (78) | 0.02 | 15 (33) | 30 (67) | 0.38 |
| Most recent partner desires fertility (*missing 3 women & 1 man)* |  | 84 (66) | 44 (34) | <0.01 | 46 (68) | 22 (32) | <0.01 |
| Communication and social pressure | | | | | | | |
| Has discussed fertility desires or family planning as a couple |  | 73 (28) | 185 (72) | <0.01 | 58 (25) | 171 (75) | <0.01 |
| Disclosed HIV status to most recent partner *(missing 1 man)* |  | 99 (34) | 189 (66) | 0.30 | 63 (27) | 174 (73) | 0.07 |
| Believes there is pressure for women to have children, regardless of HIV status |  | 91 (34) | 187 (67) | 0.13 | 60 (28) | 152 (72) | 0.80 |
| Feels pressure to have children from family or community |  | 51 (54) | 43 (46) | <0.01 | 28 (42) | 39 (58) | <0.01 |
| Discouraged from childbearing by healthcare worker when HIV status was disclosed ^b^ |  | 18 (41) | 26 (59) | 0.30 | n/a | n/a | n/a |
| Believes physician would support her decision to have (more) children ^b^ |  | 68 (38) | 109 (62) | 0.06 | n/a | n/a | n/a |
| HIV and pregnancy | | | | | | | |
| Believes HIV-positive women can give birth to HIV-negative babies *(missing 1 women)* |  | 101 (34) | 198 (66) | 0.45 | 66 (28) | 172 (72) | 0.74 |
| How ART during pregnancy affects risk of MTCT ^b^ *(missing 3 women)* | Increases | 36 (38) | 58 (62) | 0.35 | n/a | n/a | n/a |
|  | Decreases | 11 (42) | 15 (58) |  |  |  |  |
|  | No change | 58 (31) | 127 (69) |  |  |  |  |
| Believes pregnancy is unhealthy for her ^b^ |  | 45 (23) | 155 (77) | <0.01 | n/a | n/a | n/a |

ART= Antiretroviral therapy, MTCT=Mother-to-child-transmission

^a^ Percentages of participants on ART (n=273 women and n=219 men)

^b^ Questions asked of female participants only
