# Supplementary material for: Factors associated with desired fertility among HIV-positive women and men attending two urban clinics in Lilongwe, Malawi
Source: PLoS One. 2018 Jun 13;13(6):e0198798. doi: 10.1371/journal.pone.0198798 (PMC5999219; doi:10.1371/journal.pone.0198798)
Supplement: S2 Table — (DOC) [file pone.0198798.s005.doc]

S2 Table. Bivariable characteristics associated with contraceptive use at last intercourse among women and men who receive care at large, public HIV clinics in Lilongwe, Malawi and do not desire fertility

|  |  | Women* | | | Men | | |
| --- | --- | --- | --- | --- | --- | --- | --- |
|  |  | Used contraception at last intercourse | | | Used contraception at last intercourse | | |
| Characteristic |  | Yes  (n=111)  n(%) or  mean (SD) | No  (n=91)  n(%) or  mean (SD) | p | Yes  (n=125)  n(%) or  mean (SD) | No  (n=55)  n(%) or  mean (SD) | P |
| Would desire fertility if were counterfactually HIV negative |  | 55 (54) | 46 (46) | 0.89 | 49 (64) | 27 (36) | 0.22 |
| Sociodemographic | | | | | | | |
| Age |  | 32.8 (5.7) | 34.8 (6.5) | 0.02 | 38.1 (5.1) | 36.9 (5.5) | 0.14 |
| Urban residence (*missing 1 woman)* |  | 97 (54) | 13 (59) | 0.66 | 111 (70) | 47 (30) | 0.53 |
| Education: completed secondary school (*missing 1 woman)* |  | 14 (42) | 19 (58) | 0.11 | 35 (80) | 9 (20) | 0.09 |
| Religion (*missing 1 woman)* | Catholic | 24 (69) | 11 (31) | 0.08 | 25 (66) | 13 (34) | 0.58 |
|  | Not catholic | 87 (52) | 79 (48) |  | 100 (70) | 42 (30) |  |
| HIV-related |  |  |  |  |  |  |  |
| Years since HIV diagnosis (*missing 3 women)* |  | 5.0 (3.3) | 4.9 (3.6) | 0.72 | 4.3 (3.9) | 3.8 (4.1) | 0.37 |
| Currently taking ART |  | 99 (54) | 84 (46) | 0.45 | 108 (68) | 50 (32) | 0.40 |
| Health status since beginning ART^a^ *(missing 19 women and 22 men)* | Improved | 91 (54) | 77 (46) | 0.82 | 101 (69) | 46 (31) | 0.52 |
|  | Worsened | 7 (58) | 5 (42) |  | 5 (56) | 4 (44) |  |
|  | No change | 1 (33) | 2 (67) |  | 2 (100) | 0 (0) |  |
| Sexual characteristics and risk behaviors | | | | | | | |
| Married or in monogamous relationship |  | 109 (56) | 86 (44) | 0.25 | 125 (70) | 53 (30) | 0.09 |
| Most recent partner’s HIV status | Negative | 19 (53) | 17 (47) | 0.49 | 26 (72) | 10 (28) | 0.10 |
|  | Positive | 73 (58) | 53 (42) |  | 94 (71) | 38 (29) |  |
|  | Unknown | 19 (48) | 21 (52) |  | 5 (42) | 7 (58) |  |
| Length of time with current partner *(missing 7 women & 3 men)* | < 4 years | 37 (39) | 58 (61) | 0.06 | 40 (53) | 35 (47) | 0.04 |
|  | > 4 years | 101 (51) | 99 (49) |  | 116 (67) | 56 (33) |  |
| Who decides whether to use contraception *(missing 8 women & 12 men)* | Woman does | 86 (61) | 55 (39) | 0.05 | 48 (75) | 16 (25) | 0.78 |
|  | Man does or both do | 24 (45) | 29 (55) |  | 76 (73) | 28 (27) |  |
| Reproductive history | | | | | | | |
| Number of children | 0 | 1 (17) | 5 (83) | 0.04 | 0 (0) | 1 (100) | 0.41 |
|  | 1 | 10 (37) | 17 (63) |  | 10 (71) | 4 (29) |  |
|  | 2-3 | 61 (58) | 45 (42) |  | 58 (67) | 29 (33) |  |
|  | 4 or more | 39 (62) | 24 (38) |  | 57 (73) | 21 (27) |  |
| Has child born with HIV *(missing 1 woman)* |  | 28 (60) | 19 (40) | 0.45 | 19 (63) | 11 (37) | 0.43 |
| Most recent partner desires fertility *(missing 3 women)* |  | 24 (55) | 20 (45) | 0.85 | 12 (55) | 10 (45) | 0.11 |
| Communication and social pressure | | | | | | | |
| Has discussed fertility desires or family planning as a couple *(missing 1 woman)* |  | 103 (56) | 81 (44) | 0.35 | 122 (71) | 49 (29) | 0.03 |
| Disclosed HIV status to most recent partner |  | 106 (56) | 82 (44) | 0.13 | 123 (71) | 51 (29) | 0.07 |
| Believes there is pressure for women to have children, regardless of HIV status |  | 100 (54) | 86 (46) | 0.25 | 106 (70) | 46 (30) | 0.84 |
| Feels pressure to have children from family or community |  | 21 (49) | 22 (51) | 0.36 | 29 (74) | 10 (26) | 0.45 |
| Discouraged from childbearing by healthcare worker when HIV status was disclosed^b^ |  | 15 (58) | 11 (42) | 0.76 | n/a | n/a | n/a |
| Believes physician would support her decision to have (more) children^b^ |  | 65 (60) | 43 (40) | 0.11 | n/a | n/a | n/a |
| HIV and pregnancy | | | | | | | |
| Believes HIV-positive women can give birth to HIV-negative babies *(missing 1 woman)* |  | 106 (54) | 91 (46) | 0.13 | 122 (71) | 50 (29) | 0.06 |
| How ART during pregnancy affects risk of MTCT^b^ *(missing 3 women)* | Increases | 32 (55) | 26 (45) | 0.48 | n/a | n/a | n/a |
|  | Decreases | 71 (56) | 55 (44) |  |  |  |  |
|  | No change | 6 (40) | 9 (60) |  |  |  |  |
| Believes pregnancy is unhealthy for her^b^ |  | 90 (58) | 64 (42) | 0.07 | n/a | n/a | n/a |

*1 woman is missing data for contraceptive use at last intercourse

^a^ Percentages of participants on ART (n=273 women and n=219 men)

^b^ Questions asked of female participants only
